# A Long-Gap Peripheral Nerve Injury Therapy Using Human Skeletal Muscle-Derived Stem Cells (Sk-SCs): An Achievement of Significant Morphological, Numerical and Functional Recovery

**DOI:** 10.1371/journal.pone.0166639

**Published:** 2016-11-15

**Authors:** Tetsuro Tamaki, Maki Hirata, Nobuyuki Nakajima, Kosuke Saito, Hiroyuki Hashimoto, Shuichi Soeda, Yoshiyasu Uchiyama, Masahiko Watanabe

**Affiliations:** 1 Muscle Physiology & Cell Biology Unit, Tokai University School of Medicine, 143 Shimokasuya, Isehara, Kanagawa 259–1193 Japan; 2 Department of Human Structure and Function, Tokai University School of Medicine, 143 Shimokasuya, Isehara, Kanagawa 259–1193 Japan; 3 Department of Orthopedics, Tokai University School of Medicine, 143 Shimokasuya, Isehara, Kanagawa 259–1193 Japan; 4 Department of Urology, Tokai University School of Medicine, 143 Shimokasuya, Isehara, Kanagawa 259–1193 Japan; 5 Department of Otolaryngology, Tokai University School of Medicine, 143 Shimokasuya, Isehara, Kanagawa 259–1193 Japan; University of Minnesota Medical Center, UNITED STATES

## Abstract

Losses in vital functions of the somatic motor and sensory nervous system are induced by severe long-gap peripheral nerve transection injury. In such cases, autologous nerve grafts are the gold standard treatment, despite the unavoidable sacrifice of other healthy functions, whereas the prognosis is not always favorable. Here, we use human skeletal muscle-derived stem cells (Sk-SCs) to reconstitute the function after long nerve-gap injury. Muscles samples were obtained from the amputated legs from 9 patients following unforeseen accidents. The Sk-SCs were isolated using conditioned collagenase solution, and sorted as CD34^+^/45^-^ (Sk-34) and CD34^-^/45^-^/29^+^ (Sk-DN/29^+^) cells. Cells were separately cultured/expanded under optimal conditions for 2 weeks, then injected into the athymic nude mice sciatic nerve long-gap model (7-mm) bridging an acellular conduit. After 8–12 weeks, active cell engraftment was observed only in the Sk-34 cell transplanted group, showing preferential differentiation into Schwann cells and perineurial/endoneurial cells, as well as formation of the myelin sheath and perineurium/endoneurium surrounding regenerated axons, resulted in 87% of numerical recovery. Differentiation into vascular cell lineage (pericyte and endothelial cells) were also observed. A significant tetanic tension recovery (over 90%) of downstream muscles following electrical stimulation of the sciatic nerve (at upper portion of the gap) was also achieved. In contrast, Sk-DN/29^+^ cells were completely eliminated during the first 4 weeks, but relatively higher numerical (83% vs. 41% in axon) and functional (80% vs. 60% in tetanus) recovery than control were observed. Noteworthy, significant increase in the formation of vascular networks in the conduit during the early stage (first 2 weeks) of recovery was observed in both groups with the expression of key factors (mRNA and protein levels), suggesting the paracrine effects to angiogenesis. These results suggested that the human Sk-SCs may be a practical source for autologous stem cell therapy following severe peripheral nerve injury.

## Introduction

Severe nerve transection with a long gap is an irreparable injury to the living body, leading to permanent loss of related motor and sensory functions. In such cases, autologous nerve grafts have been used as the gold standard treatment [1], with the expectation of proliferation and activation of associated Schwann cells and production of neurotrophic factors and cytokines [2]. However, the prognosis is not favorable, despite the sacrifice of healthy nerves from other parts of the body.

Alternatively, scaffold bridges, which can be of synthetic or biological origin and be resorbable or non-resorbable, have been studied with the hope that bridging conduits can provide adequate mechanical support for separated nerve ends and prevent the diffusion of neurotrophic and neurotropic factors secreted by transected stumps [1]. However, it appears clear that use of these conduits alone does not facilitate nerve regeneration across long gaps [3]. Acellular conduits have also been used with several cell sources, such as Schwann cells and/or Schwann-like cells induced from cultivated bone morrow stromal cells [4], olfactory ensheathing cells [5], and adipose tissue-derived cells [6]. However, it is unlikely that these conduits could match or exceed the performance of autografts. Even the combined use of the iPS-derived neurospheres with bioabsorbable conduit transplantation showed only about 5% axonal recovery in long-gap nerve injury after 12 weeks [7].

On the other hand, we recently reported the potential therapeutic use of mouse skeletal muscle-derived multipotent stem cells (Sk-MSCs) in long-gap nerve treatment with bridging by an acellular conduit [8] based on their capacity for synchronized reconstitution of the muscle-nerve-blood vessel unit [9]. As expected, transplanted mouse Sk-MSCs actively differentiated into all peripheral nerve support cells (Schwann cells and perineurial/endoneurial cells) and blood vessel-related cells (pericytes and vascular endothelial cells), leading to 90% recovery in the number of axons and a 9-fold increase in blood vessel formation, with diminished differentiation into the skeletal muscle cell lineage [8]. Favorable functional recovery *in vivo* was demonstrated by observing subjects walk across a narrow log 8 weeks after transplantation [8]. These results were revolutionary because they showed 2–3 fold higher recoveries than the reported scores of the gold standard treatment [1].

However, it was still unknown whether human skeletal muscle also included such stem cells comparable to the mice. Human skeletal muscle-derived stem cells (Sk-SCs) have been isolated, characterized, and examined with respect to their *in vivo* differentiation capacities [10–14]. However, the therapeutic application of human Sk-SCs to nerve repair has never been investigated, even though they are thought to be involved in nerve regeneration [15]. We also studied the optimal methods for the therapeutic isolation and fractionation of human skeletal muscle-derived cells (Sk-Cs), and established appropriate cell expansion culture methods [16]. Subsequently, we demonstrated that the human Sk-Cs also exerted stem cell potential for skeletal muscle and peripheral nerve-vascular lineage cells in severely damaged skeletal muscle [16], similar to the mouse Sk-MSCs [9, 17, 18]. Therefore, it appeared that human Sk-Cs was considered also the skeletal muscle-derived stem cells (Sk-SCs).

In the present study, we first examined the differentiation capacity of human Sk-SCs in severely crushed sciatic nerves of nude rats. In order to examine the therapeutic potential of human Sk-SCs, we injected them into the long-gap transected nerve model of nude mice with an acellular conduit bridge. We strictly followed the protocol for mouse-to-mouse experiments reported previously [8]. The results clearly indicated that human Sk-SCs exerted comparable capacities to those seen with mouse Sk-MSCs [8], with regard to cell biology, tissue morphology, physiology, and therapeutic potential. It is therefore possible that combined use of human Sk-SCs and a bridging conduit is the best method for long nerve gap therapy. The present results are a significant and practical step forward in the development of a therapeutic strategy for long-gap nerve injury.

## Materials and Methods

### Human skeletal muscle samples and cell isolation

We recruited 9 patients (aged 16 to 79 years, 7 male and 2 female) whose legs were amputated after unforeseen accidents. Study protocols were carried out according to The Code of Ethics of the World Medical Association (Declaration of Helsinki), and were approved by our institutional ethics committee (Tokai University School of Medicine: BC approval No. 12I-11). All patients gave written (signed) informed consent after being a detailed explanation of the study aims and procedures. As for the patients under 20 years-old, written informed consent from the next of kin was also obtained. The consent procedures were also approved by our institutional ethics committees (see above). Muscle samples (5–10 g) were wrapped in gauze moistened with cold (4°C) physiological saline immediately after removal, and were transferred to the laboratory for isolation of stem cells within 30 minutes.

The human Sk-SCs were isolated using a previously described procedure for mouse and human muscles [8, 16, 19, 20]. Briefly, muscle samples were weighed and washed several times with DMEM containing 1% penicillin/streptomycin, and were cut into several pieces (5–7 mm thick and wide, and 40–50 mm long). Muscles were never minced. Muscle pieces were treated with 0.1% collagenase type IA (Sigma-Aldrich, St. Louis, MO) in DMEM containing 7.5% FCS with gentle agitation for 2 hours at 37°C. Extracted cells were filtered through 70-μm, 40-μm, and 20-μm nylon strainers in order to remove muscle fibers and other debris, and then were washed and resuspended in Iscove’s modified Dulbecco’s medium (IMDM) containing 10% FCS, yielding enzymatically extracted cells. Enzymatically extracted mixed cells were then prepared for staining with cell surface antigens and sorting, or were stored in liquid nitrogen with cell preservative solution (Cell Banker; Juji-field, Tokyo, Japan) until use, after pre-freezing at −80°C using a bio freezing vessel (BICELL; Nihon Freezer Co., Ltd., Tokyo, Japan).

### Sorting of enzymatically isolated cells and cell culture

**Fig 1:**
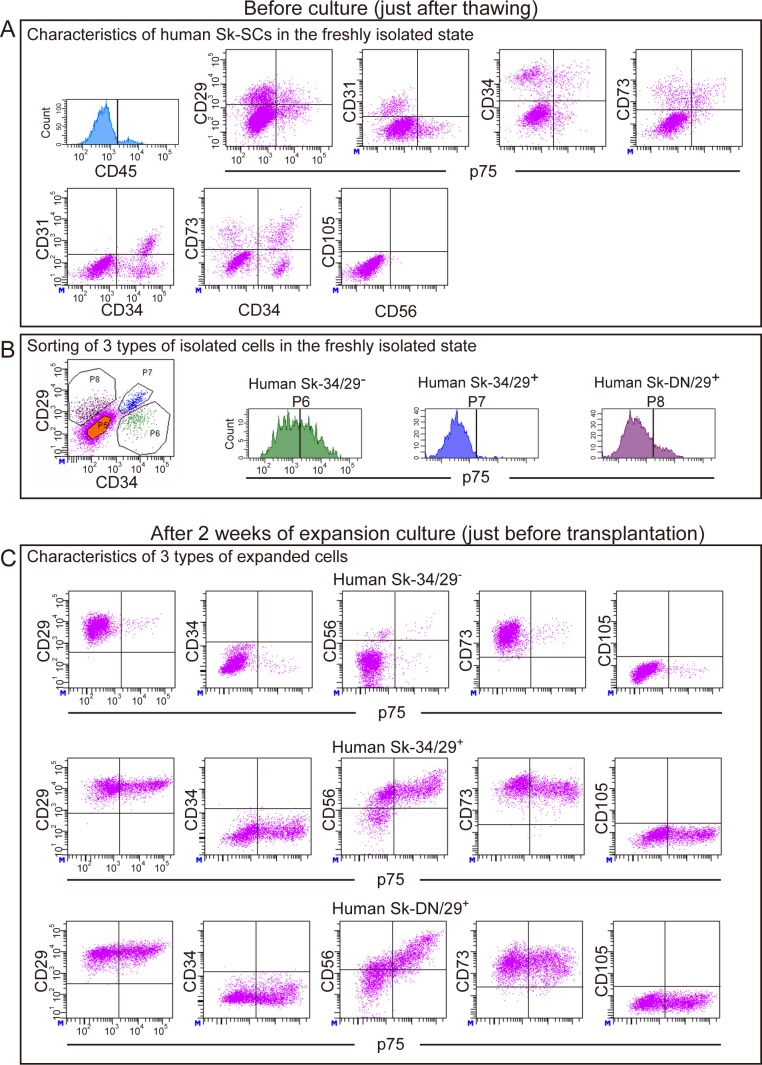
Fluorescence activated cell sorting (FACS) analysis of human Sk-SCs before and after expansion culture, and sorting of human Sk-34 and Sk-DN cells. (A) Characteristics of human Sk-SCs just after thawing, in a putative freshly isolated state. First, CD45^+^ cells were discarded as hematopoietic cells, and the remaining cells were analyzed further. Based on the results in (A), possible markers for cell sorting were CD29, CD34, CD73, and p75 (CD271). (B) We then selected CD29 and CD34, and sorted three types of cell: Sk-34/29^-^ (CD34^+^/45^-^/29^-^), Sk-34/29^+^ (CD34^+^/45^-^/29^+^), and Sk-DN/29^+^ (CD34^-^/45^-^/29^+^). We also confirmed the concentration of p75^+^ cells (see panels P6, P7, and P8). (C) Characteristics of the three types of cell after 2 weeks of expansion culture. Basically, three types of cell showed similar characteristics, while a lower distribution of p75^+^ cells was seen in Sk-34/29^-^ cells than in the initial state (see P6 in B). Note that the patterns of Sk-34/29^+^ and Sk-DN/29^+^ are similar, but that they showed quite different behaviors after *in vivo* transplantation (see Fig 1). Therefore, in our cells, FACS characteristics after expansion culture did not reflect in vivo differentiation capacity after transplantation. The validity of using CD29 in the detection of human cells (see Fig 2) is also evident (C), because of their whole positivity. Standard cell sorting was performed on all samples, and detailed FACS analysis was performed using 3 males and 1 female (ages: 17, 27, 60, and 79; muscles: soleus, gastrocnemius, and tibialis anterior).

Sorting of enzymatically isolated cells and expansion culture was performed strictly in accordance with a previous report [16]. Isolated cells were sorted using cell surface markers, including CD29 (BD Biosciences, San Jose, CA), CD34 (RAM34, eBioscience, San Diego, CA), and CD45 (30-F11, BioLegend, San Diego, CA) antibodies using a FACSAria (Becton Dickinson Japan, Tokyo, Japan), to yield CD34^+^/45^-^/29^+^ (Sk-34/29^+^), CD34^+^/45^-^/29^-^ (Sk-34/29^-^), and CD34^-^/45^-^/29^+^ (Sk-DN/29^+^) fractions. All samples were sorted accordingly. FACS analysis of human Sk-SCs before and after expansion culture as well as detailed sorting of human Sk-34 and Sk-DN cells are summarized in **Fig 1**. First, analysis was performed on freshly isolated or thawed human Sk-SCs using typical mesenchymal stem cell surface makers: CD29, CD31, CD44, CD56 (NCAM), and CD73 (purchased from BD Biosciences, San Jose, CA); CD105 and CD117 (c-kit) (from BioLegend, San Diego, CA); CD133 (from Miltenyi Biotec, Bergisch Gladbach, Germany); and CD166 (from Beckman Coulter, Brea, CA); as well as CD34 (RAM34; eBioscience, San Diego, CA) and CD45 (30-F11; BioLegend, San Diego, CA) antibodies (**Fig 1A**). Cell analysis and sorting were carried out on a FACSAria (Becton Dickinson Japan, Tokyo, Japan). Then, cells were sorted into three fractions (**Fig 1B**). Sorted cell fractions were separately cultured and expanded in the optimal conditions described previously [16], and then analyzed again (**Fig 1C**). The details of an appropriate cell culture/expansion methods (passages were limited to two times) were described previously [16].

### Recipient animals and cell transplantation

Athymic nude mice (female, BALB/cA Jcl-*nu/nu*; CLEA Japan, Tokyo, Japan, aged 5–6 wk, n = 80) and rats (male and female, F344/NJcl-*mu/mu*; CLEA, Tokyo, Japan, aged 8–12 wk, n = 17) were used as transplant recipients. Three additional mice were used to obtain normal, untreated control data. The animals were housed in standard cages and were provided food and water ad libitum. The room temperature was kept at 23 ± 1°C, and a 12:12 light-dark cycle was maintained throughout the experiment. All experimental procedures were approved by the Tokai University School of Medicine Committee on Animal Care and Use (No. 153015). All methods were undertaken to minimize potential pain and distress, and there were no animals died unexpectedly during the study.

In order to determine *in vivo* differentiation and therapeutic potential, we used two types of animal models: 1) a severe nerve crush injury model, and 2) a completely transected nerve with long-gap model. All operations were performed under inhalation anesthesia (Isoflurane; Abbot, Osaka, Japan), and body (rectal) temperature was maintained at 36 ± 1°C with radiant heat and this was maintained throughout the surgical procedure. During surgery, analgesic nonnarcotic opioid (butorphanol tartrate; 0.1mg/Kg subcutaneous infusion, Meiji Seika Pharma, Tokyo, Japan) was administered if necessary. During the recovery phase, we paid attention to animal behavior, and when pain behavior was observed, subcutaneous infusion of analgesic nonnarcotic opioid above was performed. We used the severe nerve crush injury model to simulate Seddon’s axonotmesis and/or the Sunderland’s fourth degree, which involves loss of axons, endoneurial tubes, perineurial fasciculi, and vascular networks, while continuity of the epineurium is maintained. Therefore, multiple stimulations for peripheral nerve and vascular lineage cells (such as Schwann, endoneurial/perineurial, and vascular cells) and synergistic effects of transplanted cells might be expected. Using this model, we examined the differentiation capacity of three cell fractions (Sk-DN/29^+^, Sk-34/29^+^, and Sk-34/29^-^ cells). Details of this model were described previously [8]. Briefly, the nerve crush injury model was prepared in nude rats (n = 14) as follows: the right sciatic nerve was exposed through a gluteal muscle incision. The sciatic nerve was then repeatedly crushed with forceps for a 12-mm span along the longitudinal axis. Most of the peripheral nerve support tissues were destroyed, except the epineurium, which is the outermost layer of the nerve. All transplanted cells were suspended in culture media at a concentration of 1 × 10^6^ cells/6 μl, and 3 μl of cells/nerve was injected into the destroyed hollow portion of the nerve through the remaining epineurium using a fine tip glass pipette. The same amounts of media without cells were injected as controls.

On the other hand, therapeutic capacity was examined using the complete nerve transection with long-gap model, and four transplantation groups (Sk-34 (29^+/-^, Sk-DN/29^+^, mixed [Sk-34 and Sk-DN/29^+^] and medium control) were prepared. Details of this model were also described previously [8]. For the complete nerve transection model with long-gap, the sciatic nerve was transected with a 7-mm (mouse) or 12-mm (rat) gap. The nerve gap was then bridged using an acellular conduit made from a separated esophageal submucous membrane from nude mice after 3 days of 70% ethanol dehydration. The bridging conduit was injected with three types of cell (concentration of 3 × 10^6^ cells/6 μl/nerve for rat, and 3 μl/nerve for mouse), including human Sk-34, Sk-DN/29^+^, and mixed cells (Sk-34 + Sk-DN/29^+^), or the same amount of medium alone (3 μl/nerve per mouse, and 6 μl per rat). Transplanted cells were optimally cultured and expanded with one passage, as described above. This model was also used for the RT-PCR analysis of engrafted Sk-34 cells 6 weeks after transplantation.

### Immunohistochemistry and immunoelectron microscopy

**Fig 2:**
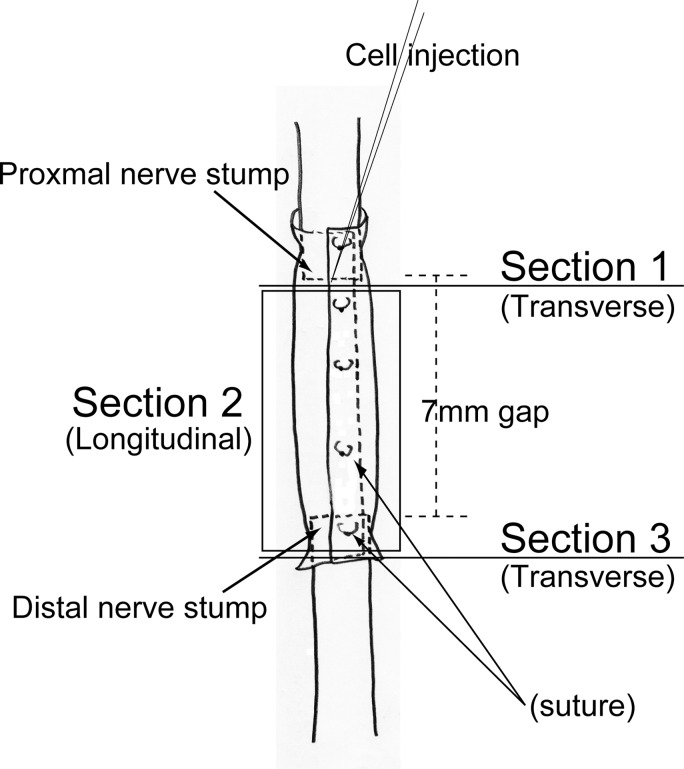
Schematic drawing of the nerve gap bridging method and extraction of histological sections. A cellular conduit was prepared using a mouse esophagus dehydrated in 70% ethanol for 3 days. Cells were injected with a fine glass pipet after suturing.

At the end of each transplantation period, recipient nude mice or rats were given an overdose of pentobarbital (60 mg/kg, i.p.), and were then perfused with warm 0.01 M phosphate-buffered saline (PBS) through the left ventricle, followed by fixation with 4% paraformaldehyde/0.1 M phosphate buffer (4% PFA/PB). Nerves were removed and fixed overnight in 4% PFA/PB, washed with graded sucrose (0–25%)/0.01 M PBS series, embedded in optimum-compound (O.C.T compound; Tissue-Tek, Sakura Finetechnical Co., Ltd., Tokyo, Japan) and frozen at − 80°C. Subsequently, in order to examine the bridged conduit, several 7-μm cross- and longitudinal sections were obtained from three portions, as shown in **Fig 2**. Section 1was a cross-section showing the proximal portion of the conduit, section 2 was a longitudinal section showing the midportion of the conduit, and section 3 was a cross-sectional profile of the distal portion of the conduit. Engrafted human cells were detected by anti-human nuclear antigen (HNA, 1:100, 4°C overnight; clone 235–1, Cy3 conjugate; Millipore, Temecula, CA). Localization of nerve fibers (axons) was determined by rabbit polyclonal anti-neurofilament 200 (N-200, 1:1000, room temperature for 1 h; Sigma, Saint Louis, MO). Myelin formation was detected by rabbit polyclonal anti-myelin basic protein (MBP; 1:200, room temperature for 2 h; Millipore, Billerica, MA). The total distribution of blood vessels was determined using rat anti-mouse CD31 monoclonal antibody (1:500, 4°C overnight; BD Pharmingen, San Diego, CA), and/or rabbit polyclonal anti-human CD31 (1:200, room temperature for 2 h; Abcam, Cambridge, UK). Immature Schwann cells were detected using rabbit anti-p75 polyclonal antibody (1:400, 4°C overnight; CST, Boston, MA) or mouse monoclonal antibody (1:100, 4°C overnight; Abcam, Tokyo, Japan), and rabbit anti-glucose transporter 1 (GLUT-1, 1:100, room temperature for 1 h; Diagnostic BioSystems, Pleasanton, CA) was used for identification of the perineurium. Myogenic cells were confirmed with rabbit polyclonal anti-skeletal muscle actin (1:300, room temperature for 1 h; Abcam, Tokyo, Japan). Reactions were visualized using Alexa Fluor-488, -594, -647-conjugated goat anti-rabbit, anti-rat, and anti-mouse antibodies (1:500, room temperature for 2 h; Molecular Probes, Eugene, OR), respectively. Nuclei were counter-stained with DAPI. Histological photographs were taken with a fluorescence multi focal projection system using Stereo Investigator (mbf Bioscience, MicroBrightField. Inc., Williston, VT) and fluorescence microscopy (Olympus BX61 with U-HGLGPS and BX-UCB, Tokyo, Japan). Staining of p75 and CD31 was further confirmed by confocal imaging. Confocal imaging was performed with a Zeiss LSM 700 confocal microscope equipped with a 40x or 40x LD “C-Apochromat” water immersion objective lens (Carl Zeiss, Jena, Germany).

For immunoelectron microscopy, sections were stained using anti-HNA (1:50, 4°C overnight; clone 235–1, biotin conjugate; Millipore), followed by HRP-conjugated streptavidin secondary antibody (1:200, room temperature for 1 h; DAKO) in order to label engrafted human cells. Reactions were visualized with DAB after fixation in 1% glutaraldehyde/0.1 M phosphate buffer. Visualized sections were then fixed in 1% osmium tetroxide/0.05 M phosphate buffer, and were prepared for electron microscopic analysis. Cell differentiation into Schwann cells, perineurial/endoneurial cells, vascular endothelial cells and pericytes, and fibroblasts were mainly observed. Immunoelectron microscopy was performed as described previously [8, 9, 16, 17, 19, 21]. Consequently, the HNA-positive cells were detected as cells having dark nuclei because of products that reacted with DAB (black dots) in the immunoelectron micrograph.

Histological analyses were performed on all samples, and 4 samples were prepared for immunoelectron microscopy.

### RT-PCR

In order to test the putative cell differentiation capacity, the expression of specific markers for skeletal muscle, peripheral nerve, and vascular cell lineages, as well as the neurotrophic and vasculogenic factors, was examined by RT-PCR of expanded (passage 1) Sk-34, Sk-DN/29^+^ cells before and after transplantation. Specific primers for human cells and analyzed materials are summarized in **S1 Table**. Expanded cells were lysed and total RNA was purified using a QIAGEN RNeasy Micro Kit. First-strand cDNA synthesis was performed with an Invitrogen SuperScript III system using a dT30-containing primer (see above), and specific PCR (35 cycles of 30 s at 94°C, 30 s at 60–65°C, and 2 min at 72°C) was performed in a 15-μl volume containing Ex-Taq buffer, 0.8 U of ExTaq-HS-polymerase, 0.7 μM specific sense and antisense primers, 0.2 mM dNTPs, and 0.5 μl of cDNA. To analyze cells after transplantation, operated nerves almost 15 mm in length (mouse) or 25 mm (rat) were detached, including the bridging conduit portions, and three nerves (mouse) or one nerve (rat) were prepared for cell isolation using the same protocol as for Sk-SCs isolation with collagenase-type IA solution (see above). Total extracted cells were then sorted using human-specific CD29 (BD Biosciences) in order to re-isolate the engrafted human cells and discard contaminated mouse derived-cells. CD29 selected almost 100% of the expanded pre-transplantation cells (see Fig 1). Sorted CD29^+^ cells were analyzed for human-specific mRNA expression. The CD29^-^ cell fraction was also prepared for the same human-specific RT-PCR, in order to confirm the inclusion of unlabeled human cells. The analysis was repeated three times with different samples (mouse, n = 9; rat, n = 3), and the relative expression intensity of each band was classified into three levels qualitatively, as an apparently strong (+3), an apparently low (+1), intermediate (+2), or undetectable (0) band with respect to the housekeeping control gene (β-actin), which was used for every electrophoresis. Obtained values were averaged.

### Protein analysis

In order to examine the paracrine effects of Sk-34 and Sk-DN cells, key cytokines related to vascular regeneration were also analyzed by an antibody array kit (Proteome Profiler Array, Human angiogenesis array kit, ARY007, R & D Systems, Minneapolis, MN) a protein levels. Cell culture supernatants of Sk-34 and Sk-DN/29^+^ cells, prepared for use just prior to transplantation, were obtained after centrifugation, and 500 μl of the supernatants were prepared for the protein analysis. Culture medium containing 20% FCS was also prepared as the negative control. The relative protein expression levels of the selected angiogenesis-related cytokines, including Ang (Angiogenin), IGF binding protein (BP)-3, IL-8, MCP-1 (monocyte chemotactic protein-1), MMP-9, PlGF (placenta growth factor), and VEGF, were determined.

### Functional assessment of downstream muscles

The most prominent functional recovery markers for long-gap sciatic nerve transection, tetanic tension outputs of the solo plantaris (PLA) and combined soleus (SOL) + gastrocnemius (GAS) muscles of nude mice were measured in both the left (non-operated control side) and right (operated side) legs and compared among the Sk-34 (n = 22), Sk-DN/29^+^ (n = 19), mixed (n = 13), and media control (n = 14) groups. Measurements were performed *in situ* under inhalation anesthesia (Isoflurane; Abbot, Osaka, Japan), and body (rectal) temperature was maintained at 36 ± 1°C with radiant heat throughout measurement. The distal tendons of reference muscles and sciatic nerves (about 15 mm) on both sides were carefully exposed, and tissues were coated with mineral oil to prevent them from drying and to minimize electric-noise interference. The details of setting and method of the tension measurement were described previously [8]. Tetanic tension output was considered to be the total recovery of nerve-muscle units, and the recovery ratio was determined based on the following equation: y = 53.848In(x)– 73.467, based on the relationship between tension output and body mass from the pooled data of non-operated control mice (n = over 120).

### Characterization of downstream tibial nerve and planter flexor muscle recovery

In order to further examine the recovery of the downstream nerve-muscles, axons of the tibial nerve were stained with N-200 associated with laminin (anti-laminin). The nerve branches associated with the muscle spindles in the plantaris muscle were stained and quantified for comparison among the Sk-34, media, and normal control groups. Similarly, myosin ATPase staining (preincubation at pH 4.2~4.3 and specifically stained for Type-I fiber) was performed for the plantaris muscles of each group in order to examine pathological changes. These include fiber type grouping, which is typically observed after denervation-reinnervation, denervation induced muscle fiber atrophy characterized by irregular fiber diameters, and ill-defined fiber colors, which indicate denervated or regenerating muscle fibers.

### Quantitative analysis

Regeneration of axons, myelin, and blood vessels in whole cross-sections of the bridging conduit was determined by the number of positive reactions to anti-N-200, -MBP, and -CD31, respectively. Reacted cells/tissues were counted using a Stereo-investigator (MBF Bioscience, MicroBrightField, Inc., Williston, VT). The analysis was performed on 4–5 sections per sample, and values were averaged. The number of axons in the downstream tibial nerves was also quantified. Values are expressed as means ± SE. Differences in morphological and functional data between groups were evaluated by parametric Tukey-Kramer post-hoc multicomparison test, and the level of significance accepted was sat at p < 0.05.

## Results

### Differentiation capacity of human Sk-SCs in the crush injury model

**Fig 3:**
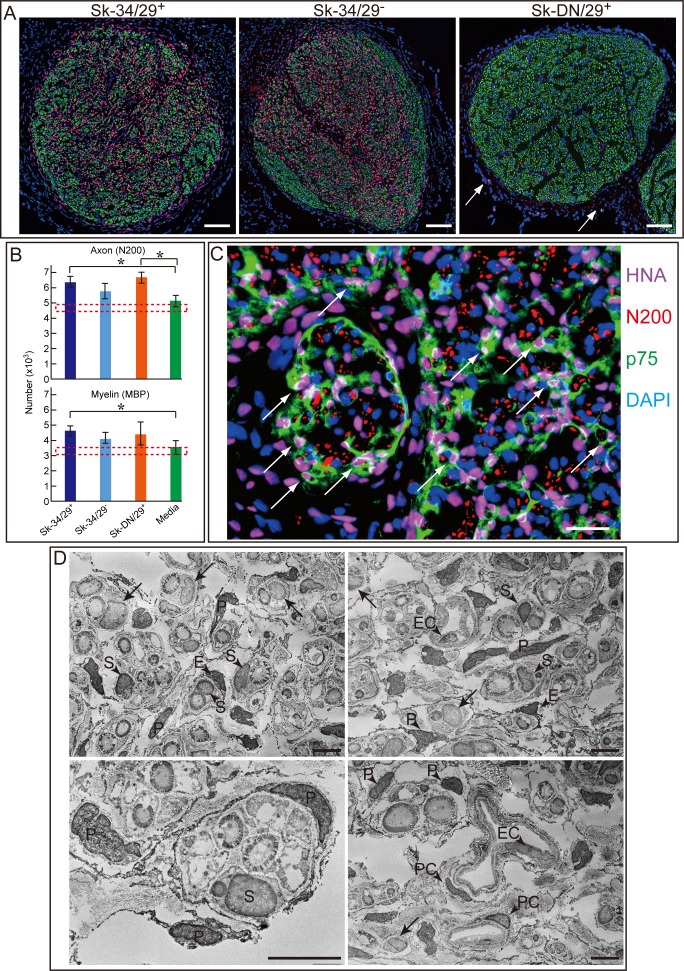
Differentiation potential of Sk-34/29^+^, Sk-34/29^-^, and Sk-DN/29^+^ cells in a severely crushed nerve niche. Results were obtained from a nude rat experiment, after transplantation of a male patient sample (age 62, tibialis anterior). (A) Cross-sectional profiles of crushed nerves 6 weeks after surgery stained with HNA (human cell nuclei, pink) and N200 (axon, green). (B) Comparison of the number of axons and myelin in the whole nerve cross-sections of four experimental groups. Dotted square shows the average range of normal non-operated control values. Complete recovery of both axon and myelin was observed in the media group, suggesting that this crush injury model is a possible self-recovery model. *P < 0.05. (C) Triple staining with HNA (purple), N200 (red), and p75 (immature Schwann cells, green) in Sk-34/29^+^ cell-transplanted nerves, showing donor human cell-derived Schwann cells (arrows). Blue = nuclear staining with DAPI. (D) Immunoelectron microscopic detection of engrafted Sk-34/29^-^ cells. Note that arrows shows HNA^-^ nuclei, and HNA^+^ nuclei show darker staining because of their DAB reaction products (higher electron density of heavy metal-binding; compare nuclei with arrows and arrowheads). Human Sk-34/29^-^ cells differentiated into Schwann cells (S), perineurial cells (P), endoneurial cells (E), endothelial cells (EC), and pericytes (PC). Note that opposite pattern of immunostaining and immunoelectron microscopy of Sk-34/29^-^ and Sk-34/29^+^ cells were presented in the S1 Fig, and confirmed that the same differentiation capacity of both cells. Bars in A = 200 μm, B = 50 μm, and C = 10 μm.

In order to determine the basic differentiation capacities of human Sk-SCs in a damaged nerve-specific niche, we first performed three types of expanded (passage 1) cell transplants in the sciatic nerve crush injury model of nude rats, using Sk-DN/29^+^, Sk-34/29^+^, and Sk34/29^-^ cells. Six weeks after transplantation, vigorous and successful cell engraftment was observed in the Sk-34/29^+^ and Sk-34/29^-^ fractions (pink nuclei), but cell engraftment was unsuccessful in the Sk-DN/29^+^ cell transplantation (**Fig 3A**). HNA^+^ nuclei (pink) were densely and uniformly distributed in both Sk-34/29^+^ and Sk-34/29^-^ cell transplanted nerves, similar to N200^+^ axons (green, **Fig 3A**). Only a few HNA^+^ cells could be seen outside of the epineurium in Sk-DN/29^+^ cell-transplanted nerves (**arrows in Fig 3A**). Transplanted cells did not interfere with nerve regeneration. Instead, the transplanted groups tended to show greater regeneration than that of the medium control (**Fig 3B**). However, the medium control group showed sufficient recovery to the normal intact levels, thus showing that this crush injury model is a self-recovering model.

With regard to the differentiation of engrafted human Sk-34/29^+^ cells, double staining of HNA^+^ nuclei and p75 was evident, indicating differentiation into Schwann cells (arrows in **Fig 3B**). Cell localization and differentiation of Sk-34/29^+^ cells was further confirmed by immunoelectron microscopy (**Fig 3C**). Cells having HNA^+^ nuclei (dark nuclei with black dots) differentiated into myelinated Schwann cells, endoneurial cells, perineurial cells, vascular endothelial cells, and pericytes. Consequently, these results indicated that Sk-34/29^+^ cells were able to differentiate into peripheral nerve support cells and vascular cells. Note that this differentiation could be attributed to Sk-34/29^-^ cells, and this is the reason that we combined Sk-34/29^+^ and Sk-34/29^-^ cells into Sk-34 cells for the subsequent experiments. In addition, these trends correspond to those observed in previous mouse Sk-MSC and human Sk-SC experiments [8, 16].

### Expression of specific mRNAs before and after transplantation of Sk-DN and Sk-34 cells

**Fig 4:**
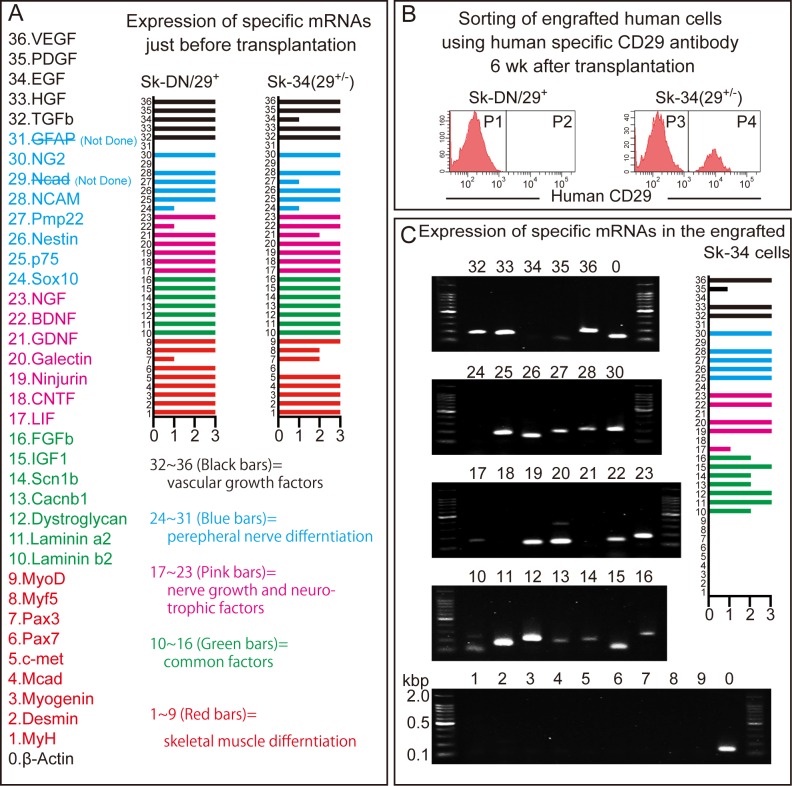
Relative expression of mRNAs specific for peripheral nerve, vascular, and skeletal muscle lineages before and after transplantation of Sk-34 and Sk-DN/29^+^ cells. Results were obtained from a nude rat experiment. (A) Expression of specific mRNAs of expanded Sk-34 and Sk-DN/29^+^ cells just before transplantation. Numbers (1–36) and colors (black, pink, green, red) in bars correspond to factor names and numbers (1–36). (B) Sorting of cells from nerves treated with bridging conduits using human cell-specific CD29 antibody 6 weeks after transplantation. Non-reactivity of human CD29 to the rat cells were preliminary confirmed. There were no human cells present in Sk-DN/29^+^ cell-transplanted nerves, but numerous cells were seen in the Sk-34 transplanted nerves. (C) Expression of specific mRNAs in sorted (engrafted) Sk-34 cells. Disappearance of skeletal muscle-related markers is clear (No.1–9). Expression was evaluated based on a housekeeping control (β-actin) provisionally as follows: 0 = none, 1 = apparently low, 2 = intermediate, 3 = apparently strong (relative to β-actin = 2), and averaged across five samples (4 males and 1 female; age: 16, 17, 32, 50, and 62; muscles: gastrocnemius and tibialis anterior).

In order to further evaluate the putative capacity for cellular engraftment and differentiation of Sk-DN/29^+^ and Sk-34 cells, the relative expression of specific mRNAs was examined before and after transplantation into the bridging conduit of a long nerve gap model in nude rats. Thirty-six markers were selected in relation to cellular commitment, growth, and regeneration of peripheral nerves, blood vessels, skeletal muscle, and common factors (**Fig 4 and S1 Table**). Just before transplantation, similar expression of specific mRNAs was observed in both expanded Sk-DN/29^+^ and Sk-34 cells, whereas the latter were Pax7 negative (**Fig 4A**). Six weeks after bridging and transplantation, operated nerves associated with the conduit were removed and prepared for cell isolation, as described for muscle cell isolation (see Methods). Isolated cells were then sorted by human-specific CD29 to obtain human-derived cells, and CD29^+^ cells were prepared for analysis of human-specific mRNAs. Notably, no reactions were observed in the same RT-PCR analysis for the CD29^-^ cell fraction, showing that almost no human cells remained. In this analysis, there were no cells detected in the Sk-DN/29^+^ cell transplantation, whereas a large number of cells were determined Sk-34 by cell sorting **(Fig 4B),** corresponding to the results of the crush injury model experiment (see **Fig 3**). In addition, sorted human-derived engrafted cells showed a lack of expression of skeletal myogenic markers, while peripheral nerve and vascular marker expression, as well as common factors, were retained (**Fig 4C**). This also indicates the differentiation of human Sk-34 cells into peripheral nerve and blood vessel support cells in the bridging conduit, thus confirming the therapeutic potential of Sk-34 cells. These results clearly suggested that expression of cell surface markers and specific mRNAs just before transplantation (after expansion culture) had no relation to successful cell engraftment (see patterns in Sk-34/29^+/-^ vs. Sk-DN/29^+^ in **Fig 4A** and **Fig 1C**). However, expression of CD34 immediately after thawing and/or isolation (before culture) is an important determinant for successful engraftment.

### Quantitative analysis of regenerated axons and myelin in the bridging conduit

**Fig 5:**
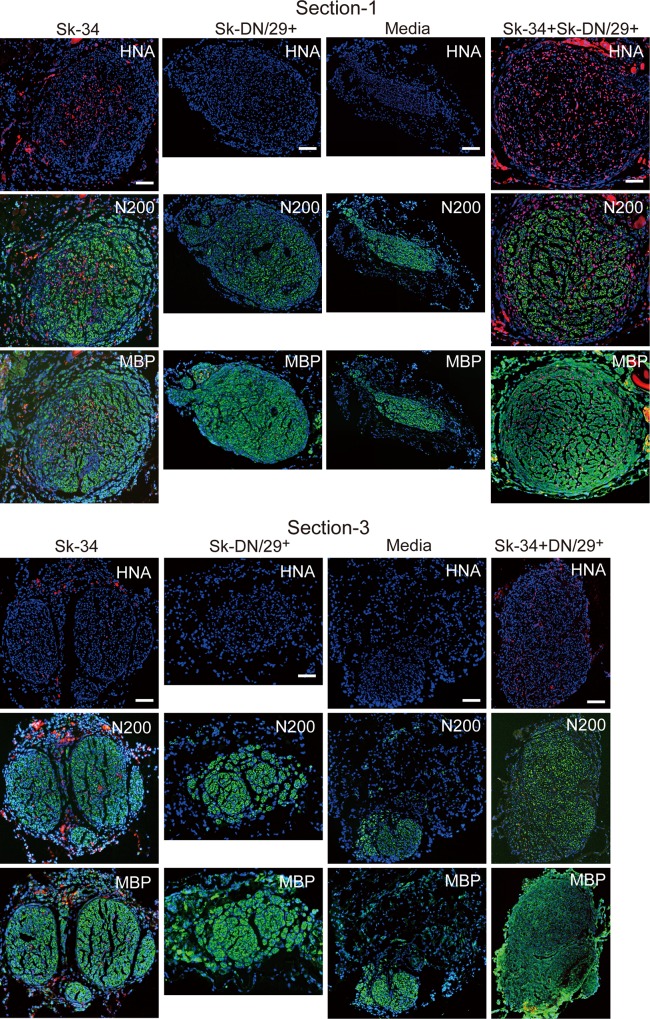
Cross-sectional profiles of bridging conduits transplanted with Sk-34, Sk-DN/29^+^, media, and mixed cells (Sk-34+Sk-DN/29^+^) 8 weeks after surgery. Results were obtained from a nude mice experiment. Upper images = section-1, lower images = section-3. A large numbers of HNA^+^ cells (red reactions) were observed limitedly in the Sk-34- and mixed-transplanted nerve (first row in both sections), with reconstitution of axons and myelin that was favorable when compared to the other two groups (green reactions in second and third row). However, there are no HNA^+^ cells in the Sk-DN/29^+^ group, but showed better axon and myelin recovery than the media group (green reactions in second and third row in columns 2 and 3). Bars = 100 μm. Data were obtained from 2 male patients (ages: 35 and 60; muscles: gastrocnemius and tibialis anterior).

In order to evaluate the contributions of transplanted cells to transected nerve recovery, regenerated axons and myelin were assessed in cross-sections 1 and 3 (see Methods, **Fig 5**). Comparisons were performed among Sk-34, Sk-DN/29^+^, mixed (Sk-34 + Sk-DN/29^+^), and media control groups, and typical features 8 weeks after transplantation are shown in **Fig 5**. Some HNA^+^ human cells were detected in the Sk-34 and mixed groups, but nothing was observed in the Sk-DN/29^+^ group. Similarly, it seems to receive the impression that favorable reconstitution of axons (N200) and myelin (MBP) was observed in the Sk-34 and mixed groups, and some recovery in the Sk-DN/29^+^ group and a slight recovery in the media group were detected (**Fig 5**).

**Fig 6:**
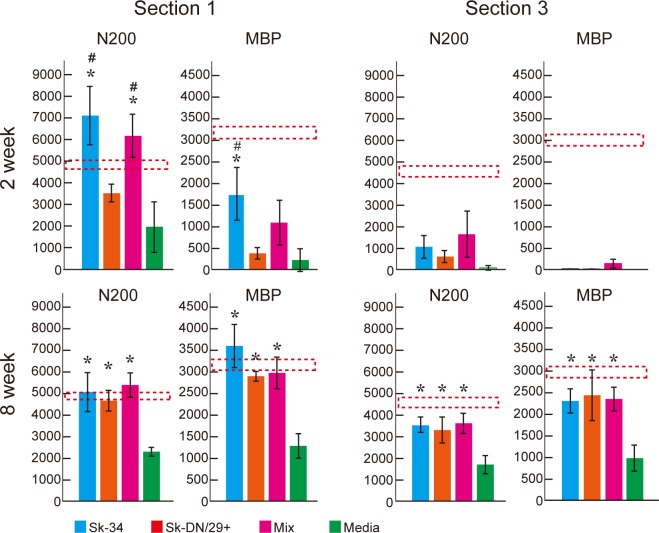
Quantitative comparison of the number of axons and myelin formation in sections-1 and -3 at 2 and 8 weeks after transplantation. Results were obtained from a nude mice experiment. Reconstitution in section-1 (proximal portion) clearly came before that in section-3 (distal portion). This was also the case for axonal regeneration and myelin formation. Note that the Sk-34 and mixed groups showed more axonal regeneration than controls in section-1 at 2 weeks, but they were both within the mean range at 8 weeks. The dotted line shows normal control values ± SE. Thus, Sk-34 group in section-1 shows 100% recoveries. *P < 0.05 (vs. media), # P < 0.05 (vs. Sk-DN/29^+^).

A quantitative comparison of axon and myelin formation in sections 1 and 3 at 2 and 8 weeks after transplantation is shown in **Fig 6**. It was evident that 1) axonal regeneration began at the proximal site of the conduit, and 2) axonal regeneration preceded myelin formation. In section 1, a temporal increase in the number of axons was observed in the Sk-34 and mixed groups 2 weeks after surgery, but a return to normal values was seen at 8 weeks. This suggests that a transient increase in axons had an accelerating effect of stem cell transplantation, but this subsequently decreased to normal levels. In contrast, a gradual increase in axons was observed in the Sk-DN/29^+^ and media groups from 2 to 8 weeks. Consequently, all three transplant groups showed 95–100% axonal recovery, but only 47% recovery was seen in section 1 in the media group after 8 weeks. With regard to myelin formation, 54% and 33% recovery was detected in the Sk-34 and mixed groups, while the other groups showed low scores at 2 weeks. At 8 weeks, however, the Sk-34 group showed 100% recovery, while 89% recovery was seen in the Sk-DN/29^+^ group, 91% was seen in the mixed group, and around 39% was seen in the media group. In section 3, around 22–35% axonal recovery was observed in the Sk-34 and mixed groups, but no myelin formation was observed at 2 weeks. However, at 8 weeks, 71–79% axonal and myelin recovery was observed in the three transplant groups, while the media group showed only about 30% recovery. Thus, taken together, the Sk-34 group showed a total of 87% axonal and 88% myelin recovery through the conduit; the mixed group showed 89% axonal and 84% myelin recovery; the Sk-DN/29^+^ group showed 83% and 84% recovery; and the media group showed 41% and 36% recovery during the 8 weeks post-transplantation.

### Quantitative analysis of early stage vascular formation in the conduit and paracrine effects of Sk-34 and Sk-DN cells

**Fig 7:**
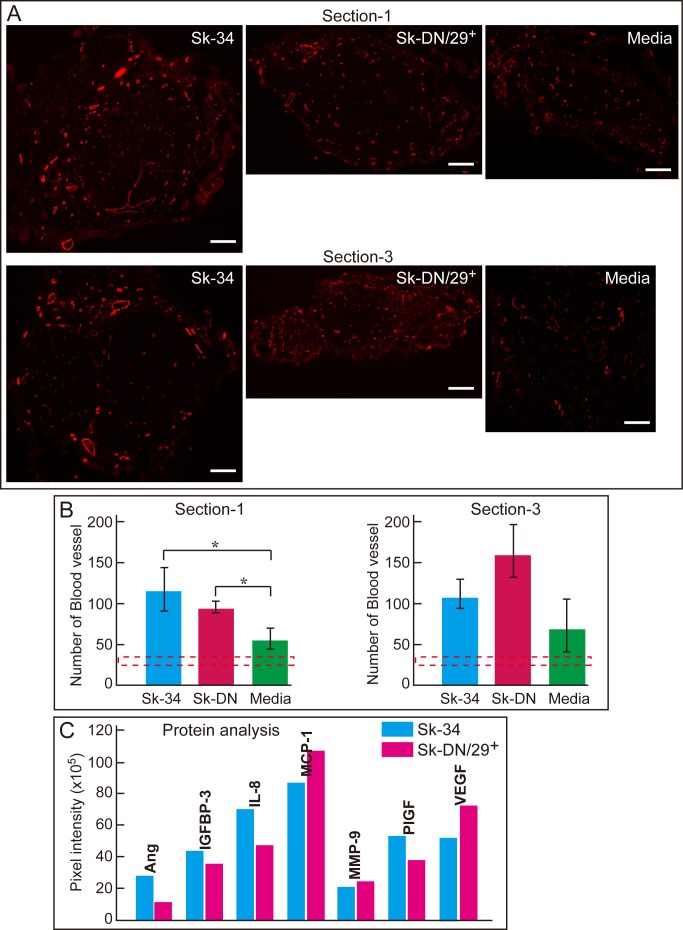
Quantitation of blood vessels formed in the conduit at 2 weeks after transplantation and the protein expression of angiogenetic cytokines. (A) Detection of newly formed blood vessels in the conduit using CD31 immunostaining in the Sk-34 (left), Sk-DN/29^+^ (center), and media (right) groups. Upper panels show Section-1 and lower panels show Section-3. Bars = 100 μm. (B) Comparison of the number of blood vessels among three groups. *P < 0.05. (C) Measurement of the protein levels of angiogenesis related cytokines. Ang: Angiogenin, IGFBP-3: Insulin-like growth factor binding protein-3, IL-8: Interleukin-8, MCP-1: Monocyte chemotactic protein-1, MMP-9: Matrix metalloprotease-9, PlGF: Placenta growth factor, and VEGF: Vascular endothelial growth factor. Levels are expressed as pixel intensity. Data were obtained from 2 male patients (ages: 35 and 60; muscles: gastrocnemius and tibialis anterior).

In order to compare the early stage vascular formation in the conduit, newly formed CD31^+^ blood vessels were assessed in cross-sections 1 and 3 (see Methods, **Fig 7**). Comparisons were performed among Sk-34, Sk-DN/29^+^, and media control groups at 2 weeks after transplantation. Typical features observed at 2 weeks after transplantation are shown in **Fig 7A**. A large number of blood vessels were formed in the conduit even at 2 weeks after transplantation (early stage of regeneration). A quantitative comparison of blood vessel formation is shown in **Fig 7B**. Two transplantation groups showed significantly higher numbers of blood vessels in Section-1 (proximal portion), and this trend was also observed in Section-3. Moreover, these three groups consistently showed a higher number of blood vessels than normal levels (dotted lines in **Fig 7B**).

Concurrently, the protein levels of angiogenesis regulatory cytokines such as Ang (Angiogenin), IGF binding protein (BP)-3, IL-8, MCP-1 (monocyte chemotactic protein-1), MMP-9, PlGF (placenta growth factor), and VEGF were measured in both expanded Sk-34 and Sk-DN/29^+^ cells prepared immediately before transplantation (**Fig 7C**). Notably, active protein expression levels of these seven factors were equally measured in both groups.

### Differentiation of human Sk-34 cells in the conduit

**Fig 8:**
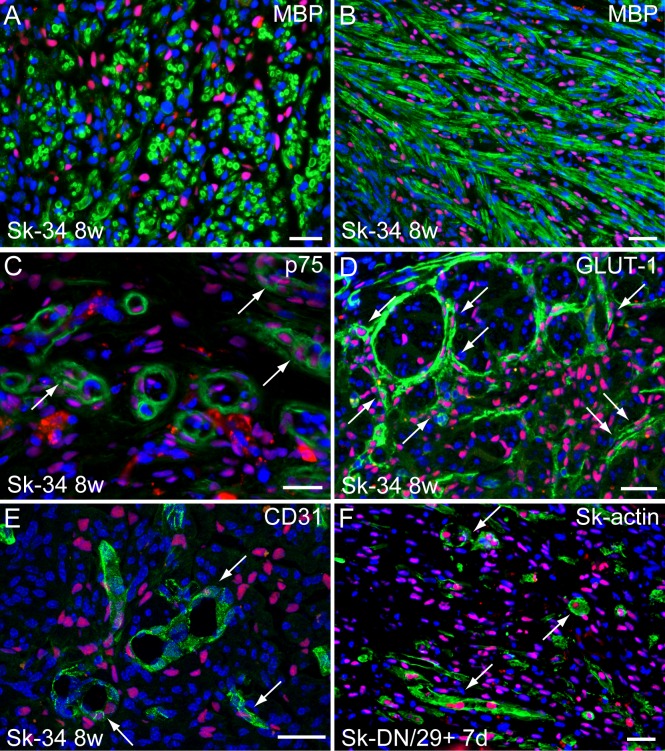
Profiles of engrafted human Sk-34 and Sk-DN/29^+^ cells in the bridging conduit. Results were obtained from a nude mice experiment. A–E = 8 weeks, F = 7 days after transplantation. Pink coloring indicates HNA^+^ nuclei of human cells. (A, B) Relationship between engrafted Sk-34 cells and myelin (MBP, green staining) formation in cross- and longitudinal-sections. (C) Engrafted human Sk-34 cell-derived, putative newly formed Schwann cells (arrows) stained with p75 (green). (D) Human Sk-34 cell-derived perineurial cells and/or perineurium stained with GLUT-1 (green, arrows). (E) Human Sk-34 cell-derived endothelial cells (CD31^+^; green, arrows). (F) Human Sk-DN/29^+^ cell-derived myotubes (skeletal muscle actin^+^; green, arrows) detected at 7 days. Blue = nuclear staining with DAPI. Bars = 50 μm. Photographs were obtained from 2 male patients (ages: 27 and 55; muscles: gastrocnemius and tibialis anterior). In addition, disappearance of Sk-DN/29^+^ cells in the bridging conduit during the 3 weeks after transplantation is confirmed in the **S2A Fig**. Response of Sk-actin in the Sk-34 cell transplanted nerve conduit is also showed in the S2B Fig. Confirmation of p75 (C) and CD31 (E) staining were further performed by confocal microscopy (**S3 Fig**). Furthermore, in the present immunohistochemistry, detection of Schwann cells and perineurium were performed by p75 and GULT-1. However, anti-p75 also react to endoneurium (endoneurial cells) and perineurium (perineurial cells), and there were confirmed by IEM (**S4 Fig**). The standard for these immunohistochemistry (N200, MBP, p75, and GLUT-1) as presented in Figs 3, 5 and 8 is shown in **S5 Fig** as for the staining for the normal control nerve.

Eight weeks after Sk-34 cell transplantation, immunohistochemical detection of Schwann cells, perineurial cells, and endothelial cells in the conduit was performed (**Fig 7**). A close relationship between HNA^+^ nuclei and myelin (MBP^+^) was observed in the cross- (**Fig 8A**) and longitudinal sections (**Fig 8B**). This relationship was further supported by evidence for differentiation into Schwann cells, which were detected by double-positive staining for p75 and HNA (**Fig 8C**, arrows). Differentiation into perineurial cells and formation of the perineurium was also demonstrated by co-staining of GLUT-1 and HNA (**Fig 8D**, arrows). Similarly, differentiation of endothelial cells was detected by double staining for CD31 and HNA (**Fig 8E**, arrows). Interestingly, several Sk-actin^+^ and HNA^+^ cells, which are considered skeletal muscle cells, were detected 7 days after Sk-DN/29^+^ cell transplantation, even in the nerve bridging conduit (**Fig 8F**, arrows). However, the number of these Sk-actin^+^ cells decreased 2 weeks after transplantation and disappeared at 3 weeks. Thus, the Sk-DN/29^+^ cells were present and/or retained in the conduit during the first 3 weeks. This disappearance of Sk-DN/29^+^ cells is shown in **S2 Fig**. Staining for the normal control nerve, as was standard for the immunohistochemistry (N200, MBP, p75, and GLUT-1) as presented in Figs 3, 5 and 8 is shown in **S5 Fig**.

### Muscle mass and tetanic tension recovery in downstream muscles

**Fig 9:**
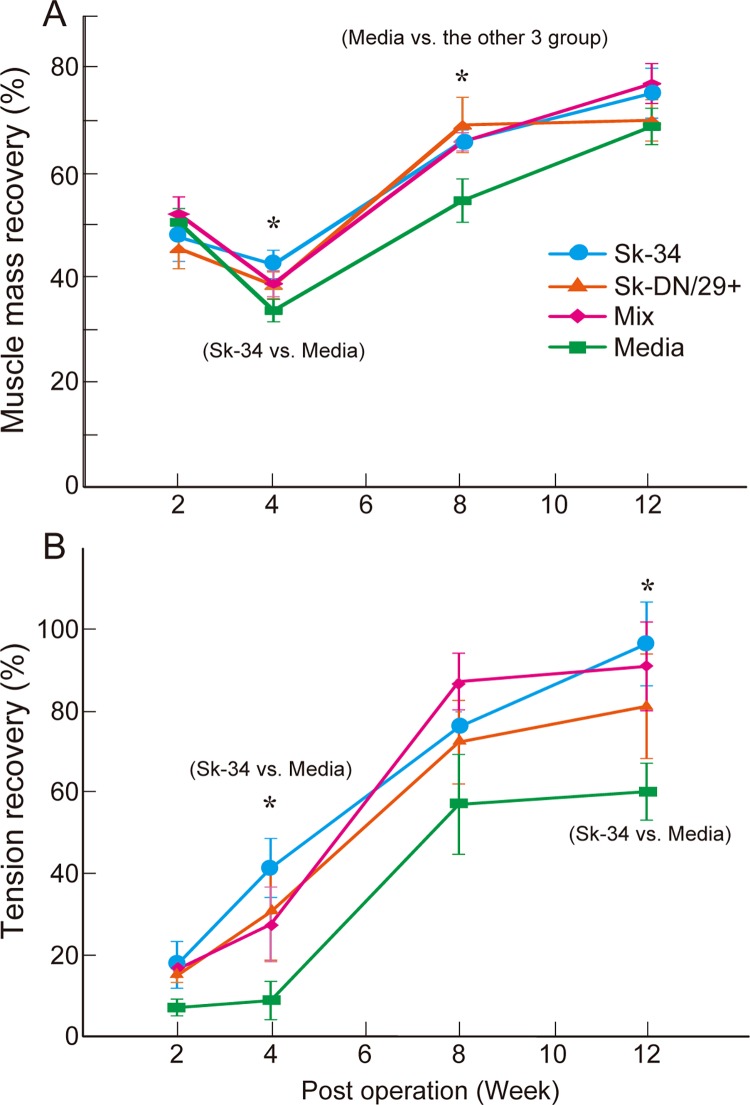
Recovery of downstream muscle mass and tension output. Results were obtained from a nude mice experiments, after transplantation of 7 patient samples (5 males and 2 female; ages: 16, 17, 27, 35, 55, 62, and 79; muscles: soleus, gastrocnemius, and tibialis anterior). (A) Changes in lower hindlimb muscle mass during the 12-week recovery phase. Values represent the total muscle mass of the gastrocnemius, plantaris, soleus, extensor digitorum longus, and anterior tibialis muscles. (B) Changes in the tetanic tension output of plantar flexor muscles (gastrocnemius, plantaris, and soleus) during the 12-week recovery phase. Mean values in each group at each stage are summarized in S2 Table. *P < 0.05.

Changes in lower hindlimb muscle mass downstream of the damaged nerve, a functional recovery marker of the transected nerve, were measured at 2, 4, 8, and 12 weeks after surgery (**Fig 9A**). Recovery was evaluated by the total mass of five muscles of the lower hindlimb plantar and dorsal flexor muscles. Changes in values of body and muscle mass and tension outputs in each group during the recovery phase are summarized in **S2 Table**. Muscle mass consistently decreased until 4 weeks after surgery in all groups, and then gradually recovered by 12 weeks. Thus, 4 weeks was considered the turning point in recovery, with a 34–43% recovery of pre-surgery mass, and significant differences were observed between the Sk-34 and media groups. At 8 weeks, the three transplant groups showed 66–69% recovery, but the media group showed a significantly lower value (55%). At 12 weeks, the Sk-34 and mixed groups showed about 75–77% recovery, while the Sk-DN/29^+^ and media groups showed around 70%.

Tetanic tension output of muscles following electrical stimulation of the sciatic nerve proximal to the gap portion was also assessed at 2, 4, 8, and 12 weeks after transplantation (**Fig 9B**). Measurements were performed in the solo plantaris and separately in the tandem gastrocnemius and soleus muscles, and were evaluated as the total function of plantar flexor muscles. In contrast to muscle mass, gradual recovery was observed in the three transplant groups, while the media group showed no changes during the first 4 weeks. After 4 weeks, all groups showed recovery that accelerated through 8 weeks, but this tended to slow by 12 weeks, except in the Sk-34 group, which showed constant recovery. Significant differences were observed at 4 weeks (41% vs. 8.8%) and 12 weeks (97% vs. 60%) between the Sk-34 and media groups. These data revealed two important points: 1) rapid recovery (that differed significantly from the media group) during the first 4 weeks in the Sk-34 group; and 2) modest recovery in the Sk-DN/29^+^ group, with transplanted cells disappearing within the first 3–4 weeks (**S1 Fig**). As a result, 97–99% tension recovery was observed in the Sk-34 and mixed groups.

### Characteristics of the tibial nerve and its branches associated with the muscle spindle in the plantaris

**Fig 10:**
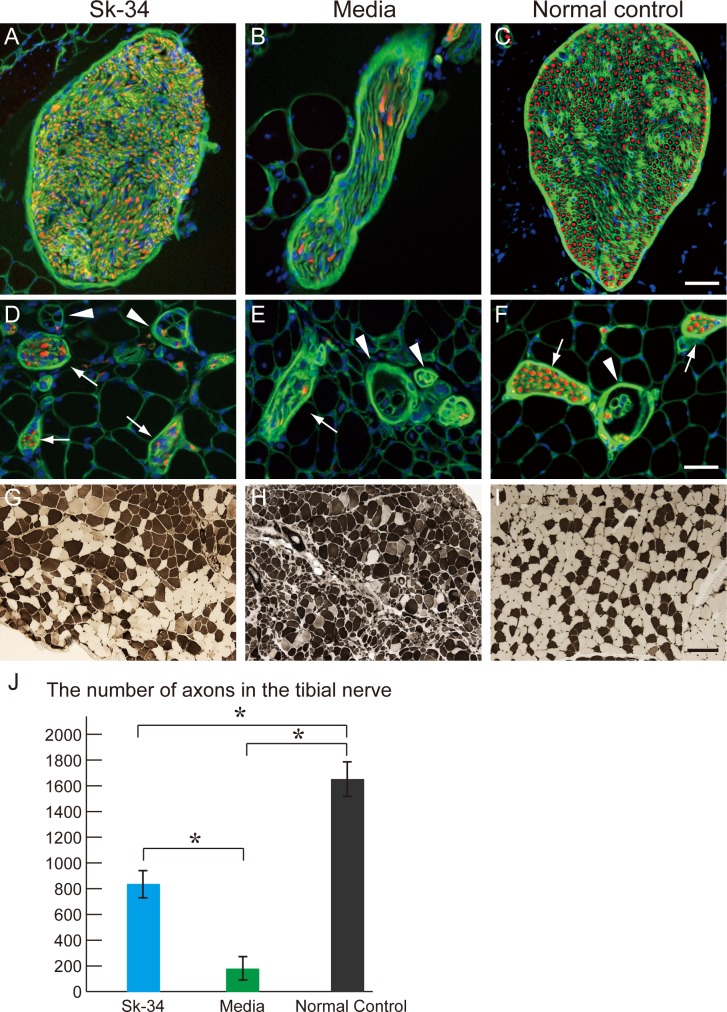
Characteristics of the tibial nerve and its branches associated with muscle-spindles in the plantaris muscle. Results were obtained from a nude mice experiment. Photographs (Sk-34) were obtained from the data of a female patient (age: 17; muscle: tibialis anterior). (A–C) Tibial nerve along with the plantaris muscle. (D–F) Tibial nerve branches (arrows) and muscle spindles (arrowheads) in the plantaris. (G-I) Portion of the gastrocnemius muscle stained with ATPase (acid) taken at the same magnification. Left column (A, D, G) = Sk-34, mid column (B, E, H) = media, and right column (C, F, I) = control. Panels A–F show staining with N200 (red) + laminin (green) at the same magnification, and blue = nuclear staining with DAPI. Bars in C, F = 50 μm, and I = 200 μm. (J) The number of axons in the tibial nerve was counted. A 50% recovery was observed in the Sk-34 group, whereas media group showed only less than 10% recovery. *P < 0.05.

Based on the above muscle mass and functional recovery data, reconstitution of peripheral nerve and muscles further from the damaged site, such as the tibial nerve and its branches associated with the muscle spindle of the plantaris, were analyzed in the Sk-34 and media groups (**Fig 10**). The ample size of the tibial nerve with the large number of axons in the Sk-34 muscle (**Fig 10A**) was almost the same as that of the control (**Fig 10C**), while in the media group, a few axons with a thin shape nerve was evident (**Fig 10B**). The number of axons in the tibial nerve was also counted in the above three groups (**Fig 10J**). Normal control showed about 1600 axons. Approximately half of this number was observed in the Sk-34 group (around 800), and the media group showed less than 200. Significant differences were observed between the three groups. A similar trend was also evident in the more downstream nerve branches located in each plantaris muscle (**Fig 10D–10F**, arrows). The media muscle tended to show fewer nerve fibers (axons) in and around the muscle spindles than the Sk-34 and control muscles (**Fig 10D–10F**, arrowheads). Furthermore, a pathognomonic irregular (small and large) fiber size was frequently observed in the media group, but was less frequently observed in the Sk-34 group (**Fig 10G and 10H**), thus confirming incomplete innervation in the media group. However, fiber-type grouping was consistently observed in both groups; thus, denervation and re-innervation clearly occurred in both groups, but innervation was incomplete in the media group and was relatively favorable in the Sk-34 group. Taken together, the results from nude mice through the present experiments ultimately bring a 50% recovery and/or retention of motor units with the reconstitution of a completely ruptured sciatic nerve, including the regeneration of the sensory nervous system such as the re-establishment of muscle spindles.

## Discussion

Recently, we established therapeutic isolation, sorting, and expansion culture methods for human Sk-SCs, and demonstrated the stem cell potency of human Sk-SCs for differentiation into skeletal muscle-nerve-blood vessel lineages after transplantation into severely damaged muscle [16]. It is possible that the differentiation capacity of human Sk-SCs is comparable to that of mouse Sk-MSCs [9, 17] because we used the identical cell isolation (enzymatic digestion and cell sorting) method. However, unlike mouse Sk-MSCs, we found that human Sk-SCs could be divided into two populations: the skeletal myogenic lineage (Sk-DN/29^+^) consisting primarily of satellite cells, and the multiple peripheral nerve-blood vessel lineage (Sk-34) [16]. Using this method, in the present study, we demonstrated that human Sk-34 cells are suitable for damaged nerve regeneration therapy. That is, transplanted human Sk-34 cells were actively engrafted into damaged sites following differentiation into Schwann cells and endoneurial/perineurial cells, and were associated with vascular endothelial cells and pericytes in the nude rat crush injury model (**Fig 3**). These results are comparable to a previous mouse Sk-MSC study focusing on severe nerve crush injury model [8]. In addition, the therapeutic potential of human Sk-34 cells with a conduit in long-gap nerve transection was also comparable to that of mouse cells [8], showing about 87% axonal and 88% myelin recovery (**Figs 5** and **6**), and favorable engraftment and differentiation into peripheral nerve-blood vessel support cells in the conduit (**Fig 8**). Similar favorable nerve reconstitution was observed in the downstream tibial nerve, which showed recovery from Wallerian degeneration. In the downstream distal hindlimb muscles, 75% of muscle mass and 97% of tetanic tension was recovered, and was associated with nearly homogeneous muscle fiber diameters (less irregularity) and innervation of the muscle spindles, suggesting sensory nerve regeneration (**Figs 9** and **10**). In addition, engrafted Sk-34 cells expressed nerve-vascular-related trophic and growth factor mRNAs 6 weeks after transplantation (**Fig 4**). The expression levels of angiogenesis-related cytokines such as VEGF in the expanded Sk-34 cells were also detected by protein analysis (**Fig 7C**), which reflects the accelerated blood vessel formation at 2 weeks after transplantation (**Fig 7A and 7B**). Furthermore, significant acceleration of morphological and functional recovery during the first 4 weeks after injury was also a characteristic of the human Sk-34 treatment associated with their active engraftment (**Figs 6**and **9**). These comprehensively improved recoveries following long nerve-gap injury have never been reported previously.

More importantly, these results were obtained from an allograft into nude mice/rats, not into SCID or NOD/SCID mice, which suggests the ease of cell engraftment. Thus, superior engraftment capacity of the present human Sk-34 cells described here might be used for autologous adult stem cell therapy. In addition, the present isolation/expansion method was confirmed to be safe setting the limit of 2 passages of cell culture, with no karyotype abnormalities appearing [16]. Furthermore, tumorigenesis *in vivo* was not observed in over 40 patient samples in over 200 transplantation experiments totally in the previous muscle experiment [16] and the present nerve experiment. Therefore, this Sk-SCs autograft method should be considered safe and practical for human nerve repair therapy.

Another group has reported similar differentiation and therapeutic capacity using human muscle-derived stem cells [15]. The isolated cells demonstrated neuronal and glial phenotypes *in vitro* and ameliorated a critical-sized sciatic nerve injury after *in vivo* transplantation into the SCID mouse model, showing differentiation into Schwann cells. The main difference from the present study is their cell isolation method, which involved a pre-plating technique using long-term cell culture with repeated passages. The recipient animals (SCID mouse), nerve injury model (less than 7 mm gap), and cell transplantation method (without a conduit) are also different. Therefore, it is difficult to compare and evaluate the therapeutic effects in both studies directly. If compared, their results of approximately 450 axons recovered in the ruptured sciatic nerve and 50% retention of downstream muscle mass are apparently less than the values of the non-cell transplanted media control group in the present study. Nonetheless, previous and present studies have collectively demonstrated that skeletal muscles are important and practical cell sources for nerve repair [9, 15–17]. Similarly, several human adult somatic stem cell lines, such as human hair follicle stem cells [22], human umbilical cord-derived mesenchymal stem cells [23], and human bone marrow stromal cells [24], have been applied to the regeneration of damaged peripheral nerves, and their cellular differentiation capacities have been examined *in vivo*. These studies consistently showed limited differentiation into Schwann cell after transplantation. Therefore, the present multiple/comprehensive contribution associated with the differentiation into all peripheral nerve support cells, along with significant contributions to angiogenesis, was never suggested. It is expected that this multiple regenerative capacity will be developed for the therapeutic use of peripheral nerve repair.

The other human skeletal muscle derived stem cells have also been isolated and characterized, and their myogenic differentiation capacity examined in vivo [10–14]. In our method, the myogenic capacity *in vivo* was limited in the Sk-DN/29^+^ cells, a fraction suggested to mainly comprise satellite cells [16], and were eliminated during the first 3–4 weeks after transplantation in the present long-gap nerve niche (**S2 Fig**). However, various cell isolation method were used, such as with or without muscle mincing, various collagenase concentrations, with or without proteases, and different cell expansion culture methods. It has been suggested that these different conditions affect the expressions of CD markers and the ability of *in vivo* engraftment and differentiation capacities of isolated cells [25]. Although a direct comparison of these cells is difficult, these studies undoubtedly suggest a remarkable potential of the skeletal muscles as a source of stem cells, for therapeutic use. Our method may have important practical implications for therapeutic use because it is safe and efficient, with rapid cell isolation and robust cell engraftment following transplantation.

Regarding the rejection of Sk-DN/29^+^ cells in the damaged nerve niche, there are several possible explanations. First, we confirmed that Sk-DN/29^+^ cells originally showed skeletal myogenic potential *in vitro* and *in vivo* [16], and that similar myogenic cell elimination in the peripheral nerve-specific niche was also confirmed in the previous mouse Sk-MSCs experiment [8]. The elimination of myogenic cells in the damaged nerve niche was also observed by another group [15]. Therefore, the myogenic cells were eliminated from the nerve-specific niche, while the expression of cell surface markers and/or specific mRNAs were similar in Sk-34 and Sk-DN/29^+^ cells just prior to transplantation (**Fig 4A** and **Fig 1**). Specifically, during the first 7 days after transplantation, Sk-DN/29^+^ cells differentiated into myocytes/myotubes producing skeletal muscle-specific proteins such as actin. However, these committed cells could not receive any further growth factors or factors promoting differentiation into mature myofibers in the damaged nerve niche, and thus, they stopped growing (differentiation arrest), underwent necrosis, and may have been phagocytized within the next 2–3 weeks (see **S2 Fig**). This trend was confirmed in myogenic cells from mouse Sk-MSCs in the damaged nerve niche [8]. Thus, we confirmed that skeletal myogenic cells cannot grow in a nerve-specific niche basically. However, we also confirmed that the present human Sk-DN/29^+^ group showed greater recovery than the media group in all comparisons (83–84% axon/myelin recovery, 70% muscle mass recovery, and 81% tension output recovery), since all other measures were clearly lower than those in the Sk-34 group. Therefore, the paracrine effects of transplanted Sk-DN/29^+^ cells during the first 3–4 weeks may have positively affected overall recovery, acting as an adjuvant for damaged nerve tissue repair. In fact, both Sk-34 and Sk-DN/29^+^ cells showed active expression of angiogenesis-regulating cytokines just before transplantation, including Angiogenin, IGFBP-3, IL-8, MCP-1, MMP-9, PlGF, and VEGF, which are potent stimulators of new blood vessel formation through the process of angiogenesis [26–33]. The presence of these cytokines in the Sk-DN/29^+^ group was associated with increased blood vessel formation in the conduit during the early stage of regeneration (2 weeks after transplantation) compared with that in the media group (**Fig 7**). This early and increased formation of blood vessels may have created a favorable oxygen-glucose environment in the nerve conduit, and resulted in significantly better recovery in the Sk-DN/29^+^ group than in the media group. This also suggests that treatment during the first 4 weeks after injury markedly affects subsequent morphological, volumetric, and functional nerve recovery; thus, it should reflect further nerve damage therapy.

In the above context, the combined transplantation of Sk-34 and Sk-DN/29^+^ cells was likely to consider the best method. This mixture was actually the same as for mouse Sk-MSCs [8], because it is difficult to divide myogenic and non-myogenic stem cells in mouse Sk-MSCs [34]. However, the results clearly indicated that synergistic effects and/or specific adverse effects were not observed in the mixed group, which showed comparable results to those of the Sk-34 group in all experiments. Thus, we conclude that mixed transplantation may be a secondary option in nerve gap therapy, in the case that an insufficient number of Sk-34 cells are obtained, and/or the case that an increased cell injection is necessary for larger nerve gaps. Therefore, the mixed transplantation of Sk-34 and Sk-DN/29^+^ cells is a possible secondary option for the nerve repair.

The 60% functional (*in situ* downstream muscle contraction) recovery in the present media control group was higher than that observed in C57BL/6N mice previously. The nude mice control group in this study showed 41% axon and 36% myelin recoveries, whereas the previous C57BL mice showed 21% axon and 23% myelin recoveries [8]. In addition, the previous functional assessment (narrow corduroy walking score) in C57BL mice indicated 34% recovery based on behavior [8]. As a result, relatively higher recoveries were observed in the nude mice media control group in this study. The reasons are unclear, but this may be a characteristic of nude mice. In addition, we must note that the basic motor ability was lower in nude mice, because they could not perform narrow corduroy walking even in the normal, non-operated control state, and this is the reason for the absence of an *in vivo* corduroy walking analysis in this study.

The material for the bridging conduit in the nerve gap is another important issue [35]. With regard to conduit materials, we used an acellular tube made from the separated esophageal submucous membranes of nude mice after 3 days of 70% ethanol dehydration, because of its size, substance permeability, and tenderness. For human therapy applications, an autologous vein of an appropriate size would be the best candidate at present, while the efficacy of cellular or acellular conditions must be examined. However, the use of an artificial tube is less invasive and much better for the patient. Biodegradable materials, such as polyglyconate [36, 37] and/or collagen type I [38] have been shown to be clinically beneficial in nerve repair, and they may be good candidate materials. However, we think that further consideration/improvement, such as adjustable size, substance permeability, and tenderness, should be necessary to complete our purpose at present.

In the present study, functional recovery of downstream muscles was apparent 2 weeks after the operation (**Fig 9B**), and Sk-34 cell transplantation resulted in significantly attenuated muscle atrophy at 4 weeks (**Fig 9A**). These results suggest that early rehabilitation can potentiate axonal regeneration, promote selective target reinnervation, and modulate central reorganization [39]. An appropriate brief electrical stimulation of the proximal nerve stump [40] and laser phototherapy [41, 42] are also candidate co-treatments for accelerating axonal growth and preventing denervated muscle atrophy.

## Conclusion

The skeletal muscle is the largest organ in the body, comprising approximately 40–50% of total body mass. Our previous study demonstrated that there were no differences in the therapeutic tissue reconstitution capacity of Sk-SCs obtained from the leg and abdominal muscles [16]. Thus, we expect that removal of a small sample (around 3 g) from the abdominal wall muscle would allow donor cells to be obtained relatively easily and safely, with minimal sacrifice. Of course, sampling around the nerve-damaged site is also possible. In addition, we confirmed the comparable differentiation and reconstitution potential of human Sk-34 and Sk-DN/29^+^ cells using MACS (magnetic-activated cell sorting). Therefore, human Sk-SCs are a potential practical source for autologous stem cell therapy following severe nerve injury.

## Supporting Information

S1 Fig Differentiation potential of Sk-34/29^+^, Sk-34/29^-^ cells in a severely crushed nerve niche. Results were obtained from a nude rat experiment, after transplantation of a male patient sample (age 62, tibialis anterior). (A and B) Triple staining with HNA (purple), N200 (red), and p75 (immature Schwann cells, green) in Sk-34/29^+^ cell-transplanted nerves, showing donor human cell-derived putative Schwann cells (arrows). Blue = nuclear staining with DAPI. (C-F) Immunoelectron microscopic detection of engrafted Sk-34/29^-^ cells. Note that arrows in C and D shows HNA^-^ nuclei, and HNA^+^ nuclei show darker staining because of their DAB reaction products (higher electron density of heavy metal-binding). Human Sk-34/29^+^ cells differentiated into Schwann cells (S), perineurial cells (P), endoneurial cells (E), endothelial cells (EC), and pericytes (PC). Note that combined results of Fig 3 and this Figure S1, showed the same differentiations of Sk-34/29^-^ and Sk-34/29^+^ cells in vivo.
(TIF)

S2 Fig (A) Disappearance of Sk-DN/29^+^ cells in the bridging conduit during the 3 weeks after transplantation. Results were obtained from a nude mouse experiment. A gradual decrease in Sk-actin^+^ (green) cells over time was evident. (B) Anti-Skeletal muscle actin staining for the Sk-34 cell transplanted nerve conduit. An adherent muscle fibers on the outside of conduit (arrows) are Sk-actin^+^, but there were no Sk-actin^+^ cells in the inside of conduit (regenerated nerve portion including transplanted Sk-34 cells) in the cross-sectional and longitudinal profiles. Arrows = accidentally adhered skeletal muscle fibers with the outside of the conduit. Red nuclei = Human Nuclear Antigen positive cells. Blue nuclei = DAPI, as the recipient cell nuclei.
(TIF)

S3 Fig Confocal images for p75 and CD31 staining. The photograph was obtained by Zeiss LSM700 confocal microscope equipped with 40x LD “C-Apochromat” water immersion objective lens (Carl Zeiss, Jana, Germany).
(TIF)

S4 Fig Immunoelectron microscopy of p75 and HNA. E = endoneurial cell, P = perineurial cell, S = Schwann cell and * = HNA^+^ nuclei. Arrows showed DAB+ reactions of the perineurium and endoneurium, and arrowheads showed DAB+ reactions of Schwann ells. (A) HNA^+^ endoneurial cells/endoneurium surrounded HNA^+^/p75^-^ Schwann cell. (B) HNA^+^ perineurial cell/perineurium surrounded several p75- Schwann cells, but one is *HNA^+^. (C) Perineurium (arrows) surrounded several Schwann cells, including *HNA^+^ and p75^+^ (arrowheads) Schwann cells. (D) Similarly, HNA^+^ perineurial cell/perineurium surrounding several p75^+^ Schwann cells (arrowheads).
(TIF)

S5 Fig Staining of N200 (A), MBP (B), p75 (C), and GLUT-1(D) in a normal control nerve. Results were obtained from a nude mouse experiment. The number of p75^+^ cells is relatively lower in a normal control nerve, because of their main containing of mature Schwann cells. Similarly, mature perineurium/endoneurium in a normal nerve does not react with GLUT-1. Squares 1 and 2 in panels A-D correspond each adjunctive panels 1 and 2, and are higher magnifications.
(TIF)

S1 Table Specific primers for human cells. bp = base pair. All primers were checked that there were never responded to the rats and mouse cells.
(DOCX)

S2 Table Statistics of body and muscle mass, and tetanic tension output in each group during recovery phase. Values are expressed as means ±S.E. TA = Tibialis Anterior, EDL = Extensor Digitorum Longus, SOL = Soleus, PLT = Plantaris, GAS = Gastrocnemius.
(DOCX)
