# Supplementary figures and images for: A Long-Gap Peripheral Nerve Injury Therapy Using Human Skeletal Muscle-Derived Stem Cells (Sk-SCs): An Achievement of Significant Morphological, Numerical and Functional Recovery
Source: PLoS One. 2016 Nov 15;11(11):e0166639. doi: 10.1371/journal.pone.0166639 (PMC5112878; doi:10.1371/journal.pone.0166639)

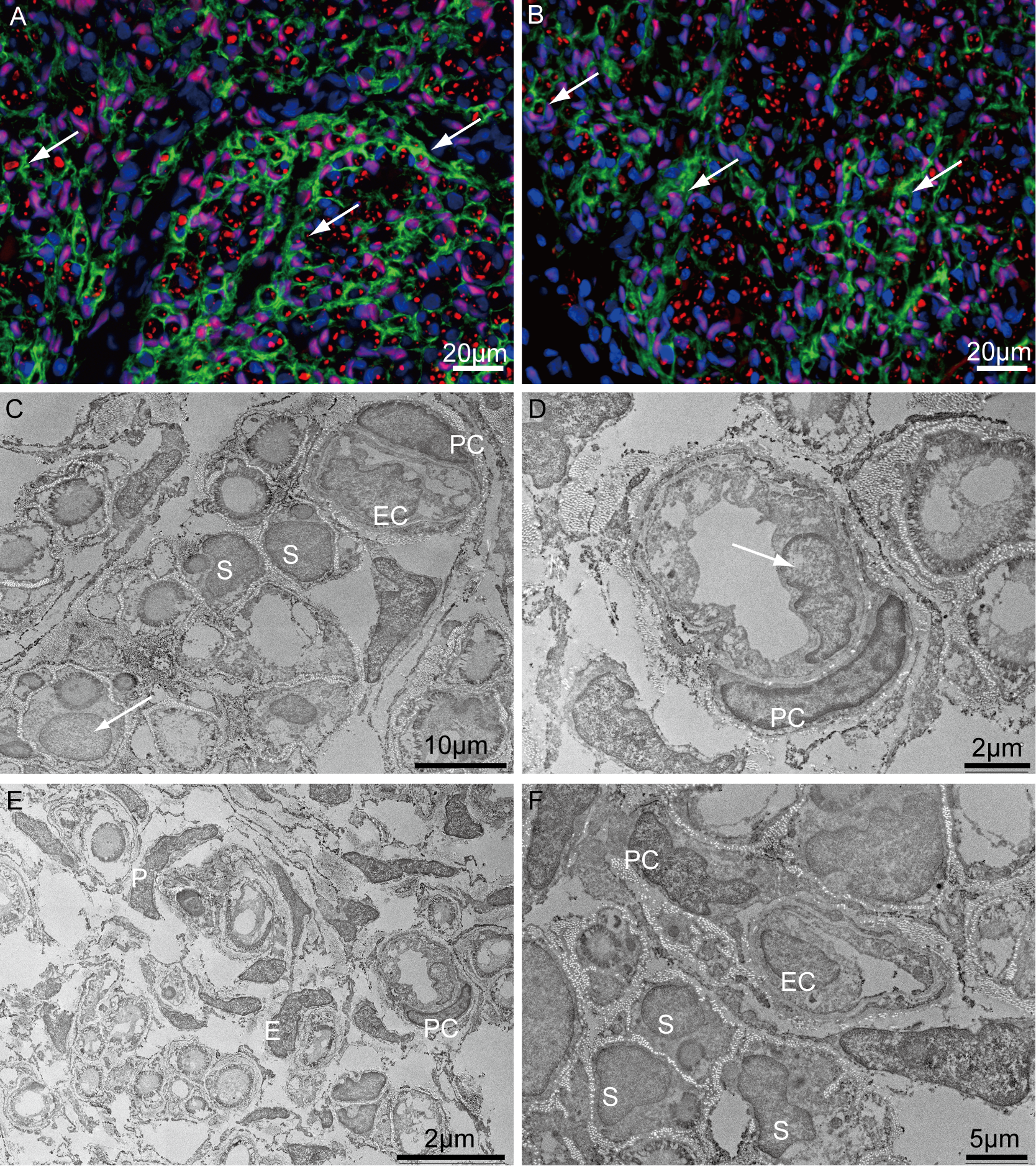

Supplement: S1 Fig — Results were obtained from a nude rat experiment, after transplantation of a male patient sample (age 62, tibialis anterior). (A and B) Triple staining with HNA (purple), N200 (red), and p75 (immature Schwann cells, green) in Sk-34/29+ cell-transplanted nerves, showing donor human cell-derived putative Schwann cells (arrows). Blue = nuclear staining with DAPI. (C-F) Immunoelectron microscopic detection of engrafted Sk-34/29- cells. Note that arrows in C and D shows HNA- nuclei, and HNA+ nuclei show darker staining because of their DAB reaction products (higher electron density of heavy metal-binding). Human Sk-34/29+ cells differentiated into Schwann cells (S), perineurial cells (P), endoneurial cells (E), endothelial cells (EC), and pericytes (PC). Note that combined results of Fig 3 and this Figure S1, showed the same differentiations of Sk-34/29- and Sk-34/29+ cells in vivo. (TIF) [file pone.0166639.s001.tif]

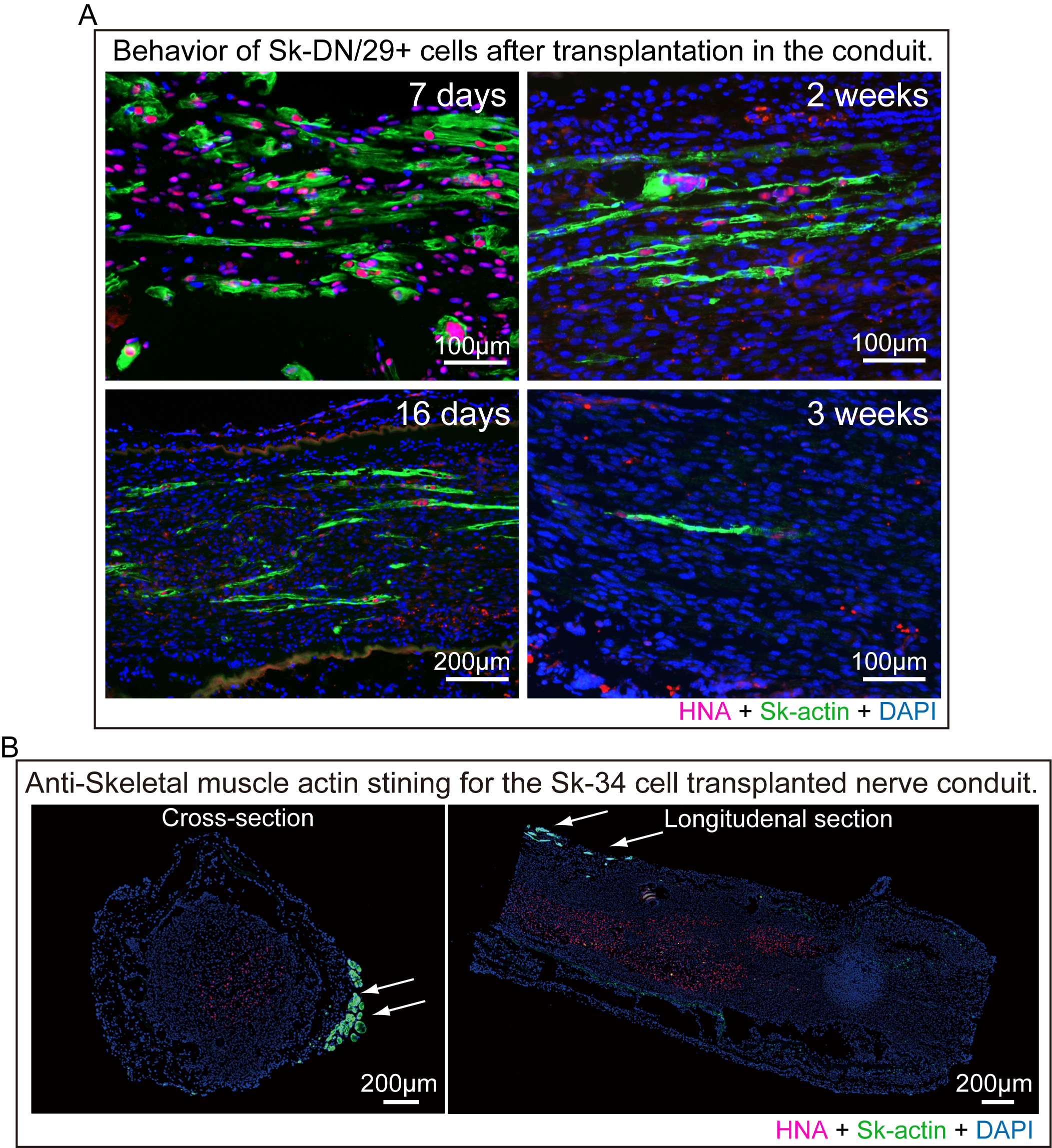

Supplement: S2 Fig — (A) Disappearance of Sk-DN/29+ cells in the bridging conduit during the 3 weeks after transplantation. Results were obtained from a nude mouse experiment. A gradual decrease in Sk-actin+ (green) cells over time was evident. (B) Anti-Skeletal muscle actin staining for the Sk-34 cell transplanted nerve conduit. An adherent muscle fibers on the outside of conduit (arrows) are Sk-actin+, but there were no Sk-actin+ cells in the inside of conduit (regenerated nerve portion including transplanted Sk-34 cells) in the cross-sectional and longitudinal profiles. Arrows = accidentally adhered skeletal muscle fibers with the outside of the conduit. Red nuclei = Human Nuclear Antigen positive cells. Blue nuclei = DAPI, as the recipient cell nuclei. (TIF) [file pone.0166639.s002.tif]

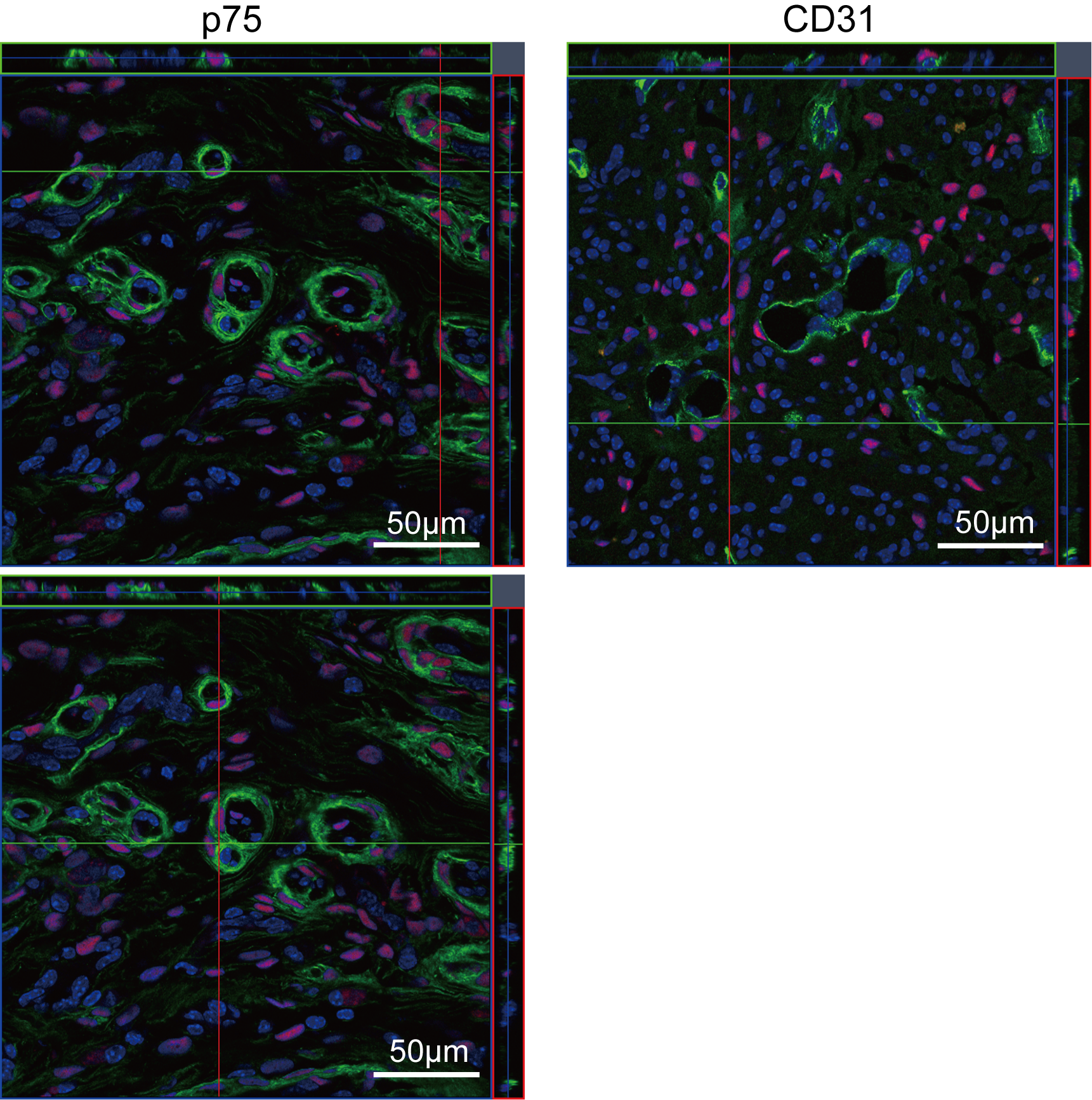

Supplement: S3 Fig — The photograph was obtained by Zeiss LSM700 confocal microscope equipped with 40x LD “C-Apochromat” water immersion objective lens (Carl Zeiss, Jana, Germany). (TIF) [file pone.0166639.s003.tif]

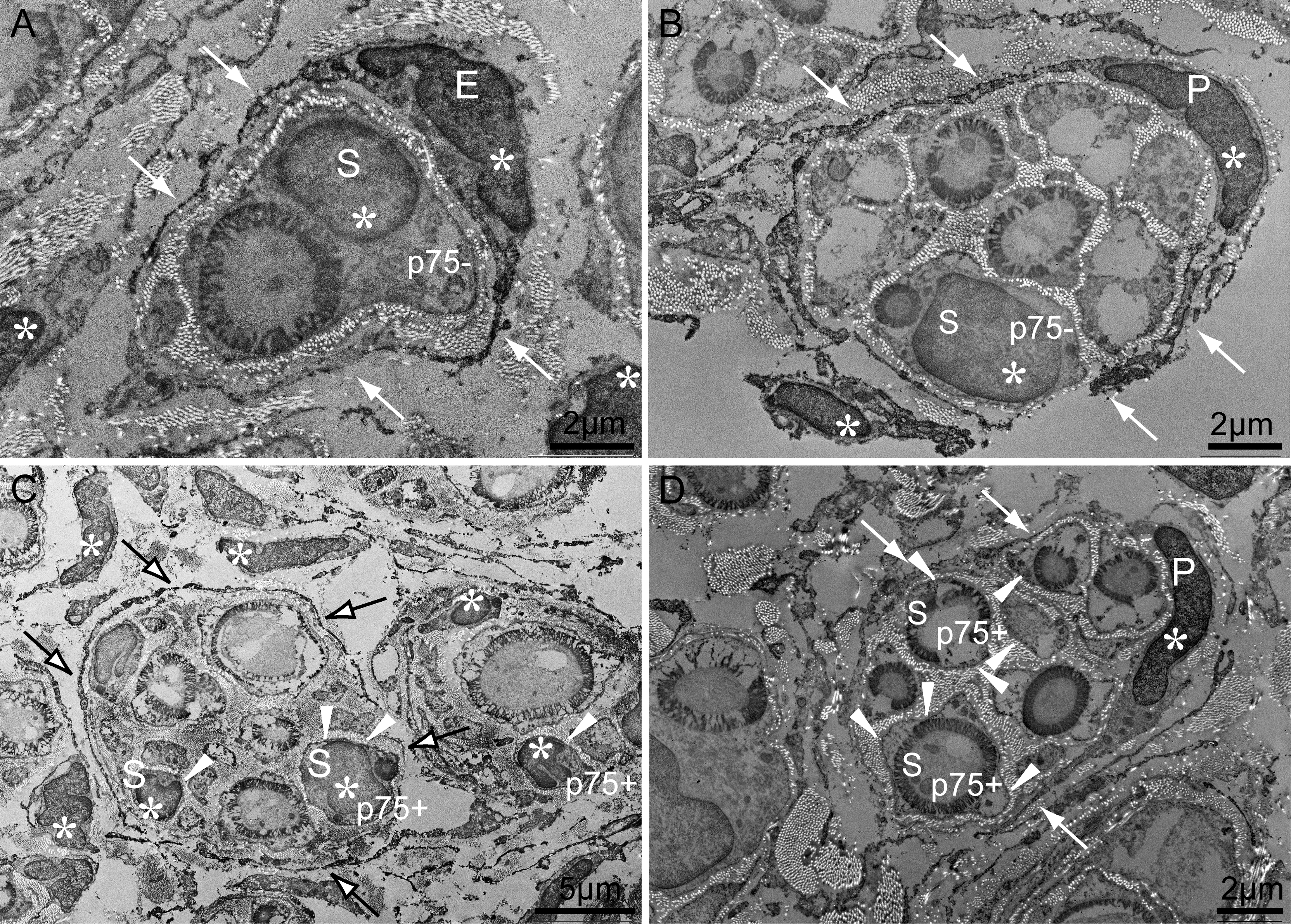

Supplement: S4 Fig — E = endoneurial cell, P = perineurial cell, S = Schwann cell and * = HNA+ nuclei. Arrows showed DAB+ reactions of the perineurium and endoneurium, and arrowheads showed DAB+ reactions of Schwann ells. (A) HNA+ endoneurial cells/endoneurium surrounded HNA+/p75- Schwann cell. (B) HNA+ perineurial cell/perineurium surrounded several p75- Schwann cells, but one is *HNA+. (C) Perineurium (arrows) surrounded several Schwann cells, including *HNA+ and p75+ (arrowheads) Schwann cells. (D) Similarly, HNA+ perineurial cell/perineurium surrounding several p75+ Schwann cells (arrowheads). (TIF) [file pone.0166639.s004.tif]

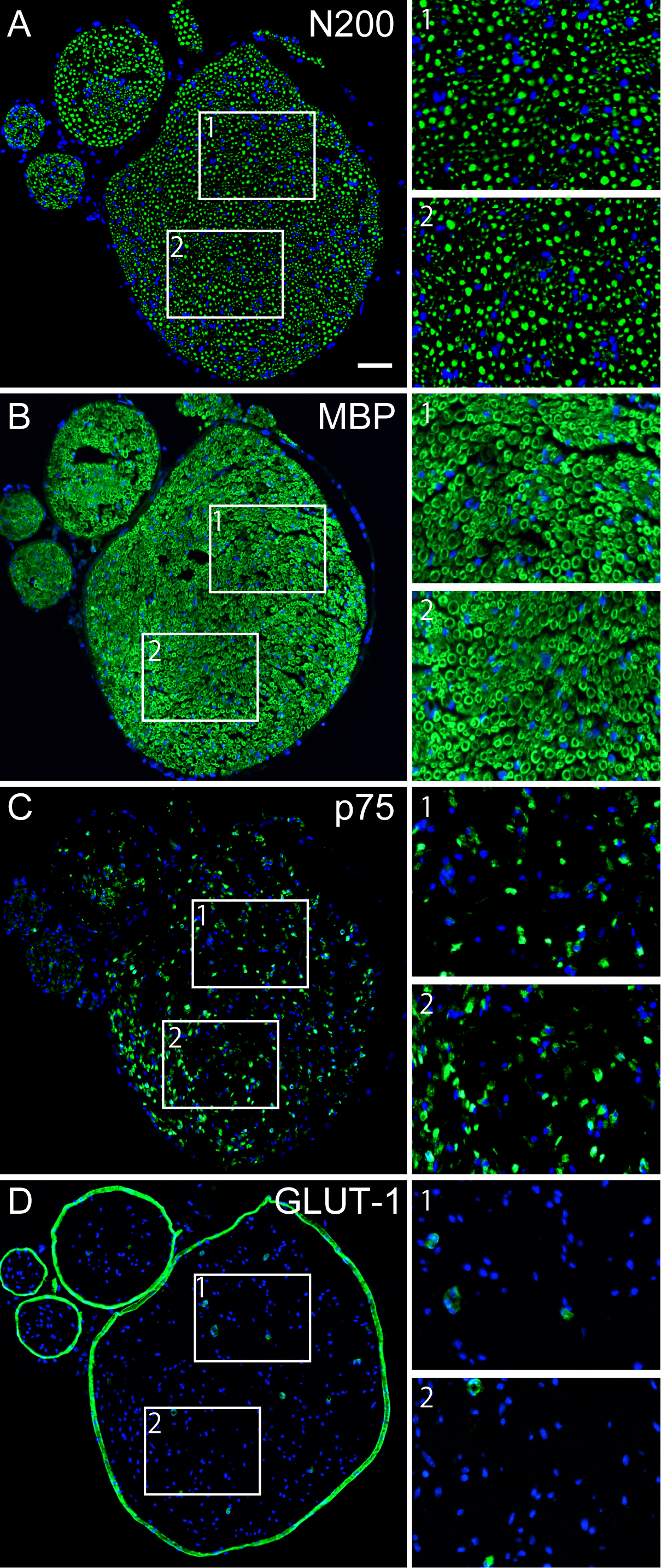

Supplement: S5 Fig — Staining of N200 (A), MBP (B), p75 (C), and GLUT-1(D) in a normal control nerve. Results were obtained from a nude mouse experiment. The number of p75+ cells is relatively lower in a normal control nerve, because of their main containing of mature Schwann cells. Similarly, mature perineurium/endoneurium in a normal nerve does not react with GLUT-1. Squares 1 and 2 in panels A-D correspond each adjunctive panels 1 and 2, and are higher magnifications. (TIF) [file pone.0166639.s005.tif]
