# Supplementary material for: A Long-Gap Peripheral Nerve Injury Therapy Using Human Skeletal Muscle-Derived Stem Cells (Sk-SCs): An Achievement of Significant Morphological, Numerical and Functional Recovery
Source: PLoS One. 2016 Nov 15;11(11):e0166639. doi: 10.1371/journal.pone.0166639 (PMC5112878; doi:10.1371/journal.pone.0166639)
Supplement: S1 Table — bp = base pair. All primers were checked that there were never responded to the rats and mouse cells. (DOCX) [file pone.0166639.s006.docx]

| Table S1. Specific primers for human cells. | | |  |  |  |
| --- | --- | --- | --- | --- | --- |
| No | Gene name (full name and/or typical role) | Product size (bp) | Forward primer | Reverse primer | Utilization purpose |
| 36 | **VEGF** (Vascular endothelial growth factor) | 200 | ACCCATGGCAGAAGGAGGAG | ACTCCAGGCCCTCGTCATTG | Vascular relating growth factor |
| 35 | **PDGF-b** (Platelet-derived growth factor-b) | 104 | GGGTTCCCTGACCATTGCTG | AGTTGGCGTTGGTGCGGTCT |  |
| 34 | **EGF** (Epidermal Growth Factor) | 367 | ATGTCCCCTGTCCCACGATG | CCAAGGTTGAGGGCAAGAGG |  |
| 33 | **HGF** (Hepatocyte growth factor) | 170 | TGCACGACAGTGTTTCCCTTC | CAGGCCTGGCAAGCTTCATTA |  |
| 32 | **TGFb** (Transforming growth factor-b) | 189 | CAATTCCTGGCGATACCTCAGC | AACCCGTTGATGTCCACTTGC |  |
| 31 | **GFAP** (Glial fibrillary acidic protein; mature Schwann cell marker) | ND | Not done | Not done | Schwann cell relating markers with myoblast (NCAM) and myelin (pmo22) markers |
| 30 | **NG2** (Protein new-glue 2 precursor; Schwann cell marker) | 124 | GGTGGGGATGGACAAATCTGG | CGTAAGGGCTTTGGTCCCATC |  |
| 29 | **N-cad** (N-cadherin, proteins that mediate calcium-ion-dependent adhesion) | ND | Not done | Not done |  |
| 28 | **NCAM** (Neural Cell Adhesion Molecule ( also known as CD56; a signal to induce neurite outgrowth via the fibroblast growth factor receptor, and myoblast marker) | 222 | CATGGCCAAGTTCTGCTGGAG | GCCCCCGAAGTACAGAATGC |  |
| 27 | **pmp22** (Peripheral myelin protein 22) | 200 | AACTCCATCTCGCCCCTTCC | TTGGGCATTTTGTCCGTGTG |  |
| 26 | **Nestin** (Nerve relate intermediate filament protein) | 155 | TGTGGCCCAGAGGCTTCTCC | TGCCGTCACCTCCATTAGCC |  |
| 25 | **p75** (neurotrophin receptor; immature Schwann cell marker) | 184 | GCACCACCGACAACCTCATC | ATGCCACTGTCGCTGTGGAG |  |
| 24 | **Sox10** (transcription factor relate to Schwann cell-development) | 253 | AGGACCCTATTATGGCCACTCG | CTGAGGTGGGCAAGGAACAG |  |
| 23 | **NGF** (Nerve growth factor) | 191 | TCCCTGCAGGACACACCATC | AAACAGCACACGGGGTGAAC | Peripheral nerve growth and trophic factors |
| 22 | **BDNF** (Brain-derived neurotrophic factor) | 154 | GCAAACATCCGAGGACAAGGTG | GGTCCTCATCCAACAGCTCTTCT |  |
| 21 | **GDNF** (Glial cell-derived neurotrophic factor) | 119 | TTGCGATGCAGCTGAGACAAC | GGTCATCATCAAAGGCGATGG |  |
| 20 | **Galectin-1** (Initial axonal growth regulator in peripheral nerves after axotomy) | 141 | CCATCGTGTGCAACAGCAAG | ATCCATCTGGCAGCTTGACG |  |
| 19 | **Ninjurin** (nerve injury-induced protein) | 137 | CATCTTCATCACGGCCTTCG | CACCTCACAGGTATGGCGACTC |  |
| 18 | **CNTF** (Ciliary neurotrophic factor) | 144 | CAGGTGCATTTTACCCCAACC | CATCCCATCAGCCTCATTGC |  |
| 17 | **LIF** (Leukemia inhibitory factor) | 169 | GCCTCCACAGCAAGCTCAAC | AGGAGTTGACAGCCCAGCTTC |  |
| 16 | **FGF2** (basic fibroblast growth factor) | 269 | GAAGAGCGACCCTCACATCAAG | TCTGCCCAGGTCCTGTTTTG | Common elements to muscle and nerve growth |
| 15 | **IGF1** (Insulin-like growth factor-1) | 174 | ACACCGACATGCCCAAGACC | GCGGTGGCATGTCACTCTTC |  |
| 14 | **Scn1b** (Sodium channel voltage gated type 1-b | 209 | CACATTGGCCGCTTCAGACAC | GGGGCAGGAGTGCATTACAGG |  |
| 13 | **Cacnb1** (Voltage-dependent L-type calcium channel subunit beta-1) | 189 | CCCGGACCCTTCAGTTGGTC | TTTCCGAGGCCGCTATTTGG |  |
| 12 | **Dystroglycan** (a central component of the dystrophin-glycoprotein complex, and possible stabilizer of sodium channel in peripheral nerve) | 217 | CGAACTGGACGACTCCAAGC | CTCCATGGGTGCTGTGAAGG |  |
| 11 | **Laminin a2** (subunit alpha 2, the major noncollagenous constituent of basement membranes) | 166 | GATGGGAACCAGGTGGAAGC | TGCCTTTGGTGAGCTTCAGG |  |
| 10 | **Laminin b2** (subunit bata-2, the major noncollagenous constituent of basement membranes) | 95 | GCGTGCTTCAAGCCATCAAC | TGTGGGGCAGTGCTAGGAAC |  |
| 9 | **MyoD** (myogenic regulatory factors) | 173 | GTGCACTCCGGTCCCAAATG | CACCACACACCATGCCTCAG | Myogenic determination and differentiation markers |
| 8 | **Myf5** (myogenic regulatory factors) | 417 | TGAGAGAGCAGGTGGAGAACTAC | GCCTTCTTCTTCCTGTGTATTAG |  |
| 7 | **Pax3** (paired box transcription factors relate to embryonic muscle development) | 215 | GCCCGGACATGTCTTGCTAAC | TTGCCAAAGCATCCATGAGG |  |
| 6 | **Pax7** (paired box transcription factors, also known as satellite cell marker) | 288 | CGGCGTTCAACCACCTTCTG | CCGGGTTCATGTGGTTGGAG |  |
| 5 | **c-met** (hepatocyte growth factor receptor) | 181 | CGGGGAAACATCCCATCAAC | TCAGCTGCAGGTATAGGCAGTGAC |  |
| 4 | **M-cad** (M-cadherin, a Ca2+-dependent cell adhesion molecule which is present in quiescent satellite cell) | 123 | GTCTGGGGGCAGAACCTGAG | GGAGGCAAAGGTGGGACTAGG |  |
| 3 | **Myogenin** (myogenic regulatory factors) | 150 | GGGGCCAAACTTTTGCAGTG | AGAGGCCCCAACCCCTTTTC |  |
| 2 | **Desmin** (Muscle relate intermediate filament protein) | 208 | AGCAGGGTGTTGGGATACTGC | CGGGGATTCCCTCTTGTAGC |  |
| 1 | **MyH** (skeletal muscle myosin heavy chain) | 324 | GGGTGGGAAGAAGCAGATCC | TCAGCAATGTCAGCCCGTTC |  |
| 0 | **β-actin** (Beta actin) | 142 | GACAGGATGCAGAAGGAGATTACT | TGATCCACATCTGCTGGAAGGT | House-keeping control gene |

bp = base pair. All primers were checked that there were never responded to the rats and mouse cells.
