# Supplementary material for: A Long-Gap Peripheral Nerve Injury Therapy Using Human Skeletal Muscle-Derived Stem Cells (Sk-SCs): An Achievement of Significant Morphological, Numerical and Functional Recovery
Source: PLoS One. 2016 Nov 15;11(11):e0166639. doi: 10.1371/journal.pone.0166639 (PMC5112878; doi:10.1371/journal.pone.0166639)
Supplement: S2 Table — Values are expressed as means ±S.E. TA = Tibialis Anterior, EDL = Extensor Digitorum Longus, SOL = Soleus, PLT = Plantaris, GAS = Gastrocnemius. (DOCX) [file pone.0166639.s007.docx]

| Table S2. Statistics of body and muscle mass, and tetanic tension output in each group during recovery phase. | | | | | | | | |  |
| --- | --- | --- | --- | --- | --- | --- | --- | --- | --- |
|  |  | 2W | | 4W | | 8W | | 12W | |
| Group |  | Physical factors | Tetanic tension N (1x10^2^) absolute | Physical factors | Tetanic tension N (1x10^2^) absolute | Physical factors | Tetanic tension N (1x10^2^) absolute | Physical factors | Tetanic tension N (1x10^2^) absolute |
| Sk-34 | Body Mass (g) | 22.8±1.1 | - | 21.8±1.1 | - | 23.3±0.3 | - | 25.0±0.9 | - |
|  | TA (mg) | 19.3±2.3 | - | 14.8±1.0 | - | 31.7±1.3 | - | 34.2±6.8 | - |
|  | EDL | 6.0±0.5 |  | 5.2±0.4 |  | 9.3±0.4 |  | 9.6±1.1 |  |
|  | SOL | 3.9±0.6 | 17.2±5.6 | 3.6±0.5 | 40.6±7.1 | 6.1±0.7 | 74.6±3.7 | 7.0±1.3 | 94.6±10.1 |
|  | PLT | 7.3±1.1 |  | 5.4±0.5 |  | 10.3±1.2 |  | 12.7±1.0 |  |
|  | GAS | 44.9±3.8 |  | 34.4±2.4 |  | 70.4±7.3 |  | 82.8±9.7 |  |
| Sk-DN/29+ | Body Mass (g) | 21.8±1.3 | - | 23.0±1.4 | - | 24.8±0.7 | - | 23.5±1.0 | - |
|  | TA (mg) | 19.4±1.9 | - | 14.9±1.7 | - | 31.6±3.7 | - | 36.7±1.7 | - |
|  | EDL | 5.9±0.5 |  | 6.0±0.9 |  | 9.1±0.8 |  | 9.9±0.9 |  |
|  | SOL | 3.7±0.8 | 14.7±1.6 | 3.3±0.2 | 30.0±11.6 | 2.7±0.8 | 70.9±10.1 | 6.3±1.3 | 79.6±12.6 |
|  | PLT | 6.4±0.6 |  | 6.0±0.5 |  | 9.7±1.3 |  | 11.5±1.2 |  |
|  | GAS | 42.1±4.0 |  | 36.4±4.5 |  | 40.9±10.3 |  | 71.8±8.1 |  |
| Mix | Body Mass (g) | 19.0±0.7 | - | 21.0±0.7 | - | 23.3±0.9 | - | 21.0±2.8 | - |
|  | TA (mg) | 17.2±1.3 | - | 15.6±1.9 | - | 28.7±1.7 | - | 28.6±3.8 | - |
|  | EDL | 5.7±0.1 |  | 5.4±0.4 |  | 8.4±0.7 |  | 8.8±1.3 |  |
|  | SOL | 3.0±0.3 | 16.1±1.4 | 3.0±0.3 | 27.0±9.0 | 4.9±0.3 | 85.5±6.8 | 4.9±0.2 | 89.2±10.7 |
|  | PLT | 5.2±0.6 |  | 6.1±0.6 |  | 10.6±0.3 |  | 9.5±0.4 |  |
|  | GAS | 39.9±1.0 |  | 33.7±1.0 |  | 65.0±3.5 |  | 68.9±7.3 |  |
| Medium | Body Mass (g) | 21.0±1.3 | - | 22.3±1.8 | - | 23.0±1.2 | - | 22.5±1.1 | - |
|  | TA (mg) | 17.3±1.0 | - | 13.6±1.6 | - | 21.3±1.7 | - | 33.3±2.4 | - |
|  | EDL | 5.7±0.4 |  | 4.9±0.5 |  | 6.7±0.8 |  | 7.5±0.9 |  |
|  | SOL | 3.2±0.4 | 7.0±2.0 | 2.5±0.6 | 8.6±4.6 | 3.7±0.8 | 55.9±12.0 | 5.8±1.0 | 59.0±6.9 |
|  | PLT | 5.0±1.1 |  | 3.9±0.1 |  | 7.0±1.1 |  | 10.4±0.8 |  |
|  | GAS | 40.9±1.9 |  | 25.9±2.4 |  | 48.0±8.4 |  | 68.3±8.4 |  |

Values are expressed as means ±S.E. TA=Tibialis Anterior, EDL=Extensor Digitorum Longus, SOL=Soleus, PLT=Plantaris, GAS=Gastrocnemius.
